# Cep57 regulates human centrosomes through multivalent interactions

**DOI:** 10.1073/pnas.2305260121

**Published:** 2024-06-10

**Authors:** Hung-Wei Yeh, Po-Pang Chen, Tzu-Chen Yeh, Shiou-Lan Lin, Yue-Ting Chen, Wan-Ping Lin, Ting Chen, Jia Meng Pang, Kai-Ti Lin, Lily Hui-Ching Wang, Yu-Chun Lin, Orion Shih, U-Ser Jeng, Kuo-Chiang Hsia, Hui-Chun Cheng

**Affiliations:** ^a^Institute of Bioinformatics and Structural Biology, National Tsing Hua University, Hsinchu 30013, Taiwan; ^b^Institute of Biotechnology, National Tsing Hua University, Hsinchu 30013, Taiwan; ^c^Institute of Molecular and Cellular Biology, National Tsing Hua University, Hsinchu 30013, Taiwan; ^d^Institute of Molecular Medicine, National Tsing Hua University, Hsinchu 30013, Taiwan; ^e^National Synchrotron Radiation Research Center, Hsinchu 30076, Taiwan; ^f^Department of Chemical Engineering, National Tsing Hua University, Hsinchu 30013, Taiwan; ^g^Institute of Molecular Biology, Academia Sinica, Taipei 11529, Taiwan

**Keywords:** liquid–liquid phase separation, centrosome, Cep57, microtubule nucleation, Cep63

## Abstract

Human Cep57 is a coiled-coil scaffold at the pericentriolar matrix (PCM), controlling centriole duplication and centrosome maturation for faithful cell division. Genetic truncation mutations of Cep57 are associated with the mosaic-variegated aneuploidy (MVA) syndrome. During interphase, Cep57 forms a complex with Cep63 and Cep152, serving as regulators for centrosome maturation. However, the molecular interplay of Cep57 with these essential scaffolding proteins remains unclear. Here, we demonstrate that Cep57 undergoes liquid–liquid phase separation (LLPS) driven by three critical domains (NTD, CTD, and polybasic LMN). In vitro Cep57 condensates catalyze microtubule nucleation via the LMN motif-mediated tubulin concentration. In cells, the LMN motif is required for centrosomal microtubule aster formation. Moreover, Cep63 restricts Cep57 assembly, expansion, and microtubule polymerization activity. Overexpression of competitive constructs for multivalent interactions, including an MVA mutation, leads to excessive centrosome duplication. In Cep57-depleted cells, self-assembly mutants failed to rescue centriole disengagement and PCM disorganization. Thus, Cep57’s multivalent interactions are pivotal for maintaining the accurate structural and functional integrity of human centrosomes.

## Significance

Cell division is a vital process in development. Errors in cell division can lead to disorders and cancer. At the heart of this process is the centrosome, a membrane-less organelle of hundreds of proteins working together to organize a bipolar spindle. It is unclear how proteins fit and function within the small confines of the centrosome. Here, we demonstrate that a crucial centrosomal protein, Cep57, self-assembles through multivalent interactions. Cep63 regulates Cep57 self-assembly. Mutations in Cep57 are linked to mosaic-variegated aneuploidy (MVA) syndrome. Our study indicates that MVA mutations impair Cep57’s ability to self-assemble. When blocking the self-assembly of Cep57 in cells, we observed abnormal structure and number of centrosomes. These findings contribute to the molecular understanding of centrosome-related diseases.

The centrosome is a membrane-less organelle functioning as animal cells’ major microtubule organizing center. It comprises a pair of perpendicularly aligned centrioles, surrounded by proteinaceous pericentriolar matrix (PCM) (1). Microtubule nucleation and organization are highly regulated by the PCM, whose function and size are cell cycle-dependent. Encircling the centriole wall, the PCM is ~300 nm thick during interphase (2–4). Cep57, Cep63, Cep152, Cep192, CDK5RAP2 (Cep215), and pericentrin are essential scaffolding proteins for PCM integrity (5–13)_._ Superresolution microscopy analyses revealed that the interphase PCM has a concentric, layered structure (2–5, 14). Cep192 and Cep57 are at the inner core surrounding the centrioles (2–5), Cep63 and Cep152 at the middle layer (3, 14), and CDK5RAP2 at the outer layer (3, 4, 14). Like a spoke, pericentrin uses its long coiled-coils to penetrate through the layered PCM structure with its N terminus protruding toward the PCM circumference (2, 4).

Before the onset of mitosis, the centriole undergoes centriole-to-centrosome conversion by recruiting more centrosomal components and expanding the PCM into a micron-sized structure (15, 16). This expansion leads to an increase in the microtubule nucleation factors, facilitating the rapid assembly of the mitotic spindle during mitosis (17, 18). It is a central question of how the PCM assembles into a dense compartment enriched with hundreds of different proteins in the human centrosome during PCM expansion. Liquid–liquid phase separation (LLPS) is a compelling concept for elucidating the organizational principles underlying membrane-less organelles. In LLPS systems, multivalent interactions through folded domains or intrinsically disordered regions drive phase separation (19–21), resulting in the formation of dynamic biomolecular condensates accessible to cognate clients (22).

Phase separation has been implicated in some of the essential centrosomal scaffolds. CDK5RAP2 functional homologs fly Cnn and worm SPD-5 self-assemble into micron-scale network structures in the absence of a macromolecular crowding agent (like PEG3350) (23, 24). Only in the presence of PEG3350 did the purified SPD-5 condensates exhibit liquid-like properties and recruit microtubule effectors TPXL-1 and ZYG-9 to facilitate microtubule nucleation (24). The human Cep63/Cep152 complex self-assembles into supermolecular architecture (25), and molecular crowding agents can induce gel-like condensate formation of this complex (26). Whether the inner layer PCM proteins phase separate to form liquid-like condensates and how human centrosomal scaffolding components coordinate to organize the centrosome remain unclear.

Located in the inner layer of PCM (5), Cep57 is a conserved protein in vertebrates that features coiled-coil domains at the N and C termini (27). It plays a crucial role in centrosome maturation throughout the cell cycle. The centrosome contains a pair of centrioles and the surrounding PCM. Cep57 maintains centriole engagement for accurate centrosome biogenesis. During mitosis, depletion of Cep57 by siRNA leads to PCM fragmentation and premature centriole disengagement (5), which licenses new centrosome formation and results in centrosome overduplication, also known as centrosome amplification. At the molecular level, mechanisms governing the Cep57-assisted centriole engagement are not fully resolved.

Cep57 is a structural hub for the centrosomal localization of Cep63-Cep152, a prerequisite for recruiting downstream PCM regulators and centriole biogenesis factors (28). During mitosis, the reduction of Cep152 and Cep63 proteins at the centrosome results in the recruitment of pericentrin through Cep57 (6). This recruitment is essential for PCM expansion and centrosome maturation (5). Understanding the mechanisms that control the PCM size is crucial for deciphering its role in physiology and diseases. Genetically, Cep57 is linked to mosaic-variegated aneuploidy (MVA) syndrome, characterized by abnormal chromosome numbers, aberrant spindle formation, and overamplified centrosomes (29–31). While extensive research has revealed the cellular functions of Cep57, the precise molecular mechanisms through which it regulates PCM organization and microtubules remain elusive.

Here, we demonstrate that purified human Cep57 undergoes a reversible liquid–liquid phase separation (LLPS) in a controlled environment. Using various tools, we identified at least three regions, the N-and C-terminal coiled-coil domains (NTD, CTD) and a polybasic LMN motif, that contribute to phase separation. Cep57 NTD and LMN motif bind to the CTD to drive higher-order complex formation. Cep57 condensates can concentrate α/β-tubulin dimers for microtubule nucleation; in counteraction, Cep63 limits the formation and microtubule assembly activity of Cep57 condensates. Blocking multivalent interactions of Cep57 by overexpressing Cep57 disease mutation or truncation mutations induced centrosome amplification. Moreover, when using siRNA targeting Cep57, the observed phenotypes, including centriole disengagement and PCM disorganization/fragmentation, cannot be rescued by Cep57 LMN or CTD mutants. Collectively, our data reveal the critical role of Cep57’s multivalent interactions in regulating PCM assembly and maintaining centriole engagement. Our findings may provide molecular insight into therapeutic strategies against centrosome-related diseases.

## Results

### Cep57 Undergoes LLPS under Physiological Conditions.

**Fig. 1.**
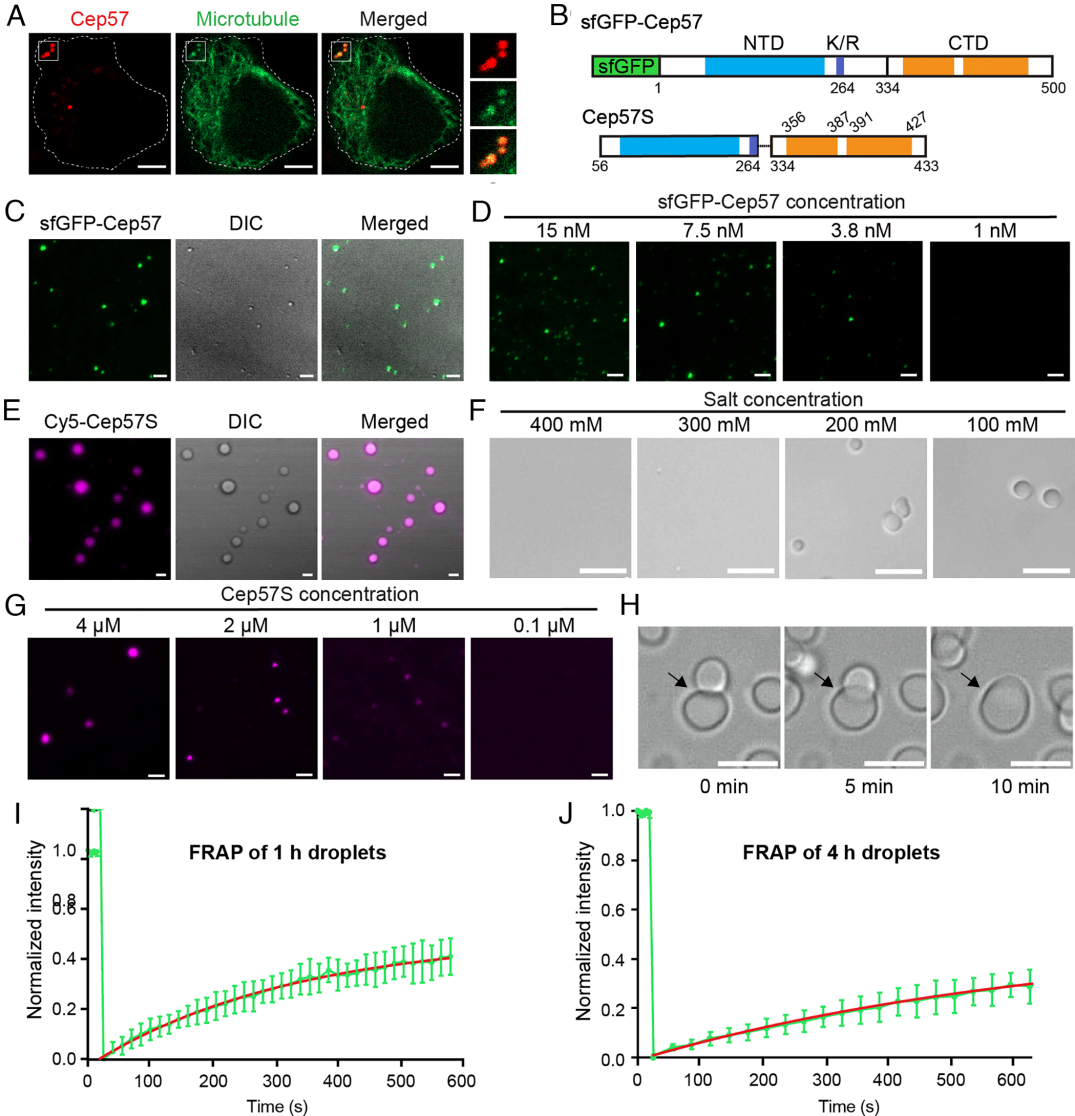
Human Cep57 and Cep57S undergo liquid–liquid phase separation in physiological conditions. (*A*) Confocal images of HeLa cells coexpressing Cep57-mCherry (red) and Neon-MAP4m as the microtubule marker (green) for 16 h. (Scale bar, 5 μm.) (*B*) Domain organization of sfGFP-Cep57 and Cep57S. Coiled-coil N-terminal domain (NTD), blue; coiled-coil C-terminal domain (CTD), orange; poly-K/R motif (K/R), dark blue. Cep57S is composed of residues 56 to 264 fused to 334 to 433. (*C*) TIRF images of sfGFP-Cep57 droplets. The sfGFP-Cep57 (25 nM) were visualized 4 h after condensation in low salt (200 mM NaCl). (Scale bar, 2 μm.) (*D*) Determination of the critical concentration for sfGFP-Cep57 condensate formation at 22 °C overnight. (Scale bar, 2 μm.) (*E*) TIRF images of Cep57S (13 μM, 5% Cy5 labeled). (Scale bar, 2 μm.) (*F*) Salt-dependent condensate formation of Cep57S (8 μM). (Scale bar, 5 μm.) (*G*) Determination of the critical concentration for Cep57S (5% D488 labeled) condensate formation at 22 °C overnight. (Scale bar, 2 μm.) (*H*) Fusion of two Cep57S droplets in 250 mM NaCl. (Scale bar, 5 μm.) (*I* and *J*) FRAP analysis of Cep57S condensates. The recovery curve of DyLight 488 (D488) labeled Cep57S droplets is shown. For the 1-h droplets (n = 7), plateau = 0.49 ± 0.01; t_1/2_ = 205.1 s ~ 252.3 s (95% CI). For the 4-h droplets (n = 11), plateau = 0.52 ± 0.04; t_1/2_ = 411.7 s ~ 690.5 s (95% CI).

The Cep57-Cep63-Cep152 interaction network plays a critical role in the centrosome cycle. The observation that the Cep63/Cep152 complex forms a higher-order structure and condensates that contribute to the centrosome’s dynamic self-assembly nature (25, 26) prompted us to examine whether Cep57 also has phase-separation property. The 14 to 18 h overexpression of Cep57-mCherry in HeLa cells resulted in spherical puncta formation in the cytoplasm (Fig. 1*A*). Some of these puncta colocalized with microtubules. Similar phenotypes were observed when a C-terminal mEGFP or N-terminal HA was fused to Cep57 (*SI Appendix*, Fig. S1*A*). Therefore, puncta formation is less likely due to the tags. After 20 h of overexpression, these constructs decorated microtubules (*SI Appendix*, Fig. S1*A*), consistent with previously reported microtubule-binding activity (13, 27). When Cep57 expression is low, it forms cytoplasmic puncta, but when it’s high, it decorates microtubules.

To examine whether Cep57 can self-assemble, we purified superfolder GFP (sfGFP) tagged Cep57 from SF9 cells through tandem affinity purification in high salt (1 M NaCl) (Fig. 1*B* and *SI Appendix*, Fig. S1*B*). We observed that sfGFP-Cep57 formed spherical droplets in micron size when the salt was reduced to 200 mM without crowding reagents (Fig. 1*C*). The spherical morphology/boundary suggests a segregated phase of sfGFP-Cep57 condensates. Moreover, the critical concentration for phase separation is ~4 nM, close to the physiological concentration of Cep57 in cells (Fig. 1*D*) (32). However, sfGFP-Cep57 was prone to degradation, resulting in poor quality and thereby impeding biochemical characterization. By removing some unstructured and less conserved regions, a shorter protein construct named Cep57S was created. This construct comprises residues 56 to 264 fused to 334 to 433, exhibiting high protein quality (Fig. 1*B* and *SI Appendix*, Fig. S1*C*). The circular dichroism (CD) spectrum of Cep57S demonstrates that Cep57S is helical, consistent with being a coiled-coil protein (*SI Appendix*, Fig. S1*D*). The formation of Cep57S condensates occurred immediately after the reduction in salt concentration (Fig. 1*E*). Cy5 labeled Cep57S was highly enriched in droplets. The round shape of Cep57S condensates indicates a liquid-like state. Given that Cep57S preserved the phase separation property of the full-length protein and has a better protein quality and purity, we used Cep57S as a surrogate to characterize the biophysical properties of Cep57 phase separation.

We performed a series of image-based assays to characterize the physiochemical properties of Cep57S condensates. First, the phase transition of Cep57S was salt-sensitive. At a fixed protein concentration (8 μM), Cep57S was highly soluble in high salt (1 M to 300 mM), while droplets were visualized in low salt conditions (200, 100 mM) (Fig. 1*F*). Moreover, the condensate formation was reversible. Cep57S droplets formed in low salt were dissolved in high salt when protein concentration remained constant (*SI Appendix*, Fig. S1 *E* and *F*). Second, Cep57S displayed a concentration-dependent phase transition with a critical concentration in the submicromolar range in the presence of 200 mM NaCl (Fig. 1*G*). Third, Cep57S was dissolved in 1,6-hexanediol, a reagent used to test phase separation by disrupting hydrophobic interaction (*SI Appendix*, Fig. S1 *G* and *H*). Furthermore, Cep57S droplets coalesced and formed larger ones over time (Fig. 1*H*). Similar to the dynamic nature of Cep57’s interacting partner Cep63 and Cep152 in cells (26), fluorescence recovery after photobleaching (FRAP) assay showed Cep57S molecules were exchanged between the bulk environment and condensates. The younger droplets (formed for ~1 h) displayed higher dynamics when compared to the older ones (formed for ~4 h). The half-time of recovery for the younger droplets was ~230 s, while it was ~550 s for the older ones (Fig. 1 *I* and *J*). The reduction of dynamics indicates the condensate becomes hardening over time. Taken together, the formation of Cep57S assembly is salt and concentration-dependent. The morphology and dynamic properties indicate that purified Cep57S undergoes LLPS in a simplified, low-salt buffer.

### Interactions of the CTD with NTD and Poly-K/R Motif Drive LLPS of Cep57.

**Fig. 2.**
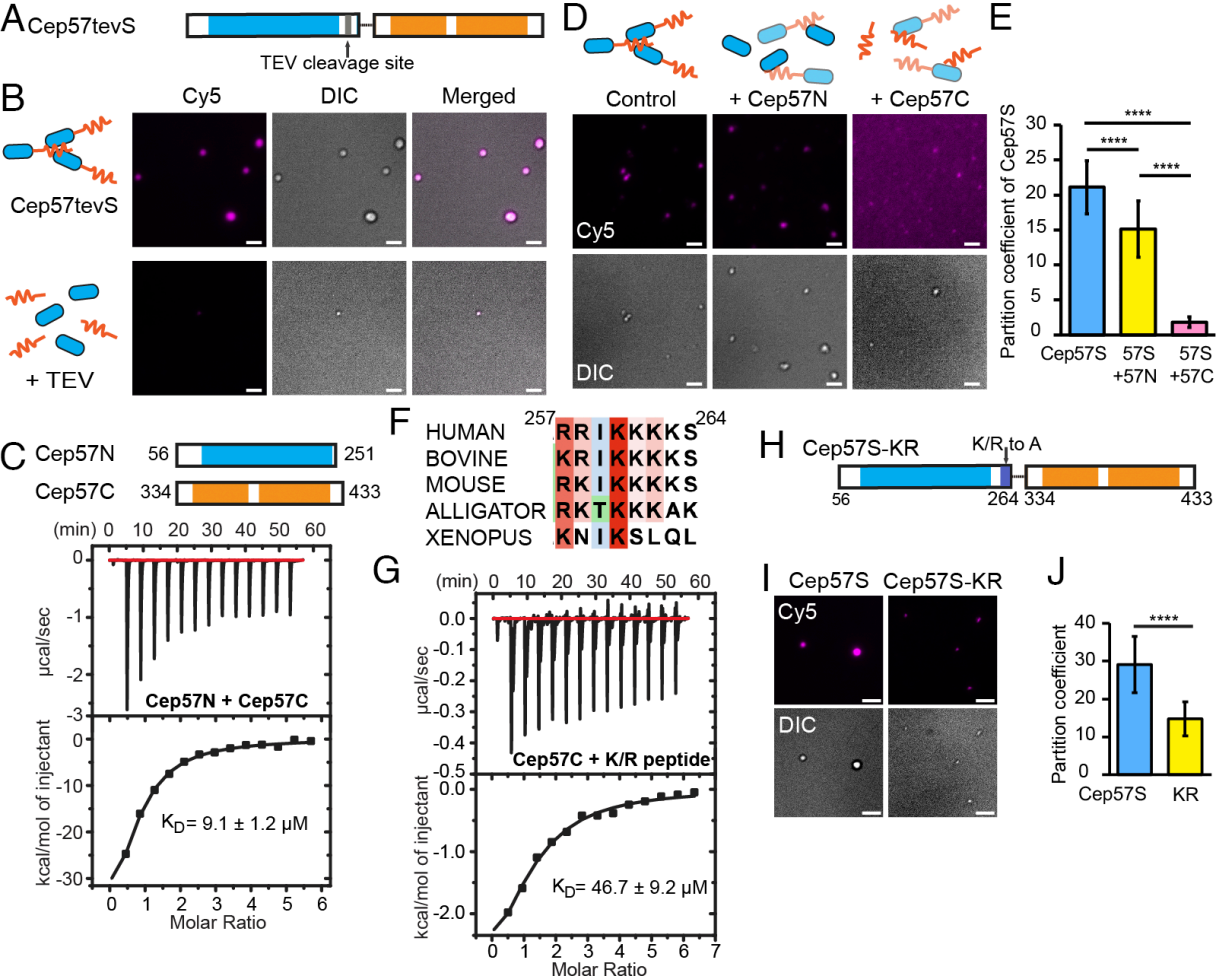
The NTD, CTD, and poly-K/R motif of Cep57 are required for LLPS. (*A*) Construct design of Cep57tevS, where a TEV cleavage site (ENLYFQG) was inserted between residue 251 and 252. (*B*) TIRF images of Cep57tevS condensates in the absence or presence of TEV protease. Cep57tevS was incubated with TEV protease at 22 °C for 6 h prior to image acquisition. Cep57tevS concentration, 4 μM (5% Cy5 labeled). Three repeats were performed for this assay. (*C*) ITC measurement of the binding affinity between Cep57N and Cep57C. (*D*) TIRF images of Cep57S in the presence of Cep57N or Cep57C. 2.5 μM of Cep57S (5 % Cy5 labeled) was incubated with 25 μM of competitors at 22 °C for 6 h prior to image acquisition. (*E*) Quantification of the Cep57S partition coefficient in experiments from panel (*D*). N = 41 (Cep57S), 51 (Cep57S + Cep57N), and 44 (Cep57S + Cep57C) pooled from three experimental repeats. (*F*) Sequence alignment of Cep57 K/R motif from human (Q86XR8), bovine (Q865V0), mouse (Q8CEE0), alligator (A0A151P2C8), and xenopus (F7C5H8). (*G*) ITC measurement of the binding affinity between Cep57C and the K/R motif. (*H*) Schematic of Cep57-KR, 6 K/R residues located from residue 257 to 264 were mutated to Ala. (*I*) TIRF images of Cep57S and Cep57S-KR (6 μM, 5% Cy5 labeled). (*J*) Quantification of partition coefficient of Cep57S proteins in experiments from panel (*I*). n = 80 (Cep57S) and 47 (Cep57S-KR) pooled from three repeats. [****] *P* < 0.0001. (Scale bar, 2 μm.)

Cep57 contains an N-terminal coiled-coil domain (NTD) and a C-terminal coiled-coil domain (CTD). To map the regions responsible for LLPS, we purified Cep57N (residues 56 to 251) and Cep57C (residues 334 to 433). Both fragments were highly soluble and displayed a monodisperse profile on gel filtration in 150 mM salt (*SI Appendix*, Fig. S2 *A* and *B*). We, therefore, speculated that both Cep57N and Cep57C are required for phase separation. To verify this, we generated a Cep57tevS construct, into which a TEV protease cleavage site was inserted between the NTD and CTD (Fig. 2*A*). In the phase separation assay, Cep57tevS presented similar LLPS potency to Cep57S as both constructs have similar critical concentrations (*SI Appendix*, Fig. S2*C*). However, after 6 h of incubation with TEV protease, the formation of Cep57S condensates was abrogated (Fig. 2*B*). The cleavage was confirmed by SDS-PAGE analysis (*SI Appendix*, Fig. S2*D*). These results suggest that the driving forces for assembling the higher-order complex of Cep57S involve the interaction between the NTD and CTD. In agreement with our speculation, the isothermal titration calorimetry (ITC) experiment showed a micromolar-ranged interaction between Cep57N and Cep57C (Fig. 2*C* and *SI Appendix*, Table S1). To assess the importance of this N–C domain interaction in LLPS, we defined the partition coefficient of Cep57 constructs as the intensity in condensate divided by that in the bulk environment. The excess of Cep57N or Cep57C decreased the partition of Cep57S in the condensates (Fig. 2 *D* and *E*), indicating Cep57N or Cep57C alone attenuates phase separation by competing for the intermolecular interactions between Cep57S molecules. A similar inhibitory effect by Cep57C was observed on sfGFP-Cep57 (*SI Appendix*, Fig. S2*E*). Hence, Cep57 and Cep57S use similar mechanisms for phase separation.

The formation of Cep57S droplets is salt dependent (Fig. 1*F*), implying that electrostatic interactions may play a role in phase separation. By sequence analysis, we identified a highly conserved K/R-rich motif located C terminally to the NTD (Figs. 1*B* and 2*F*). To examine whether this highly positively charged motif contributes to phase separation, we generated a Cep57S-KR mutant by replacing six charged Lys and Arg residues in this motif with Ala (Fig. 2*H*). This mutation significantly lowered the partition coefficient of Cep57S-KR and critical concentration for LLPS, compared with that of Cep57S (Fig. 2 *I* and *J* and *SI Appendix*, Fig. S2*F*). This mutational analysis demonstrated that this positively charged motif contributes to the phase separation of Cep57S. To map the binding site for the K/R-rich motif, we measured the affinity of the synthesized K/R peptide to different Cep57 fragments. The ITC experiments showed the K/R peptide binds to Cep57C with a K_D_ of 46.7 μM, while no interaction was detected between the K/R peptide and Cep57N (Fig. 2*G* and *SI Appendix*, Fig. S2*G*). In addition, we noticed that Cep57C exhibited a stronger inhibitory effect than Cep57N (Fig. 2 *D* and *E*) because excess Cep57C can block the NTD and K/R motif from network formation. Our data show that the NTD, K/R motif, and CTD are required for the phase separation of Cep57. Finally, we cannot rule out that the deleted regions (aa 1 to 55, 265 to 333, and 434 to 500) may contribute to phase transition, as Cep57 has a lower critical concentration for phase separation compared to Cep57S.

### Dimerization of Cep57C Is Important for LLPS.

**Fig. 3.**
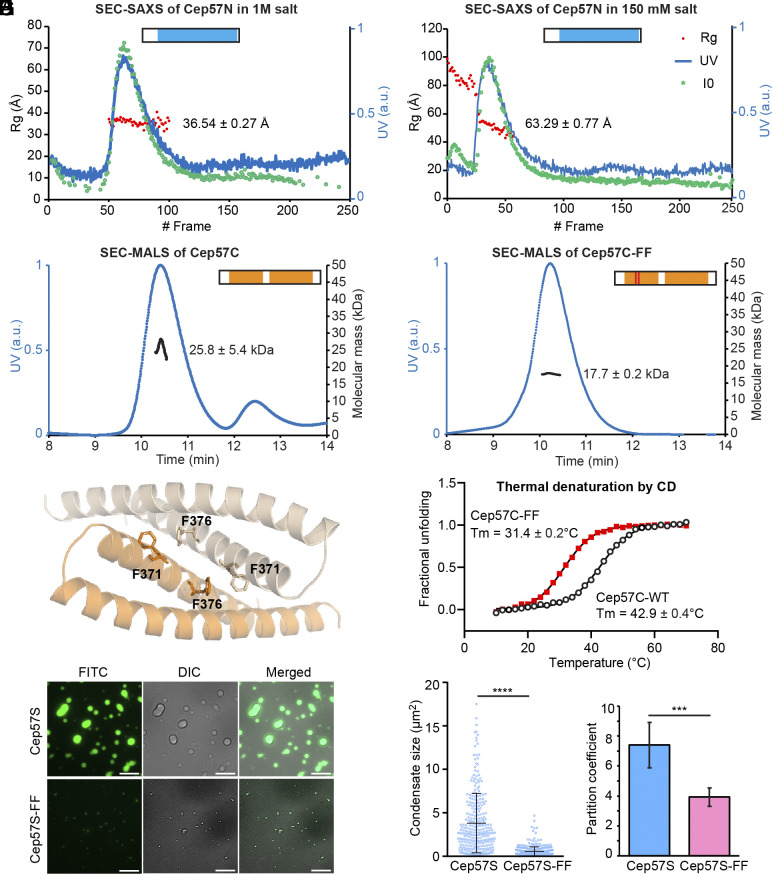
CTD dimerization facilitates phase separation of Cep57. (*A*) SEC-SAXS profiles of Cep57N in 1 M and 150 mM salt. The Rg values are as indicated. (*B*) SEC-MALS profile of Cep57C. The calculated molecular mass for Cep57C is 12.5 kDa. (*C*) Crystal structure of Cep57C shown as a dimer. The side chain of F371 and F376 are in stick presentation. (*D*) SEC-MALS profile of Cep57C-FF. (*E*) Thermal denaturation of Cep57C and Cep57-FF. (*F*) TIRF images of 15 μM (5% D488 labeled) Cep57S or Cep57S-FF in 200 mM NaCl. (Scale bar, 10 μm.) (*G* and *H*) Quantification of the condensate size and the partition coefficient of Cep57S or Cep57S-FF in experiments from panel (*F*). n = 279 (Cep57S) and 420 (Cep57S-FF) pooled from three repeats. [***] *P* < 0.001; [****] *P* < 0.0001.

Coiled-coil domains often self-assemble into high-order oligomers and have been shown to promote phase separation (33). Here, we examined the oligomeric state of the NTD and CTD. By SEC-SAXS (size exclusion chromatography coupled small angle X-ray scattering) analyses, Cep57N displayed an Rg of 36.5 Å with an estimated molecular mass (Mr) around 33.4 kDa in high salt (1 M) and an Rg of 63.3 Å with an estimated Mr around 54.4 kDa in low salt (150 mM) (Fig. 3*A*), while the calculated size for a monomer is 26 kDa. Thus, Cep57N is a dimer in physiological conditions. Cep57C is also a dimer in 150 mM salt based on the analysis of SEC-MALS (size exclusion chromatography coupled with multiangle light scattering). The estimated Mr (25.8 kDa) is two times larger than the monomer (12.5 kDa) (Fig. 3*B*).

To obtain atomic structure information on Cep57, we determined a crystal structure of the Cep57C at 2.10 Å resolution in the space group of P6_4_22 (*SI Appendix*, Table S2). The Cep57C adopts a helix–turn–helix fold, with one protomer in the asymmetric unit, forming a symmetric dimer (Fig. 3*C*). The ordered residues span from 354 to 428, with Helix 1 comprising residues 356 to 387, and Helix 2 comprising residues 391 to 427. The dimer interface is composed of two universally conserved F371 and F376 in Helix 1 (Fig. 3*C* and *SI Appendix*, Fig. S3*A*), whose benzyl rings pack well in a near 90-degree orientation with the centroid distance of 5.6 Å, a very stable π–π stacking interaction (34). To test whether dimerization is important for LLPS, we performed structure–function analyses by replacing F371 and F376 with Ala in Cep57C and Cep57S, referred to as Cep57C-FF and Cep57S-FF. SEC-MALS data showed that the size of Cep57C-FF mutant decreased to ~17 kDa, indicating it is a mixed population of dimer and monomer (Fig. 3*D*). Furthermore, the FF mutation reduced the thermal stability of Cep57C by 11.5 degrees (Fig. 3*E*), while the secondary structure remains similar to the wild-type Cep57C as monitored by circular dichroism (*SI Appendix*, Fig. S3*B*). Therefore, FF mutation weakens the dimer formation. Next, we investigated the role of CTD dimerization in phase separation. Compared with the wild-type Cep57S, the FF mutation decreased the condensate size, partition coefficient, and critical concentration for LLPS (Fig. 3 *F*–*H* and *SI Appendix*, Fig. S2*F*). Therefore, Cep57C dimerization is essential to promote Cep57 condensation.

### Cep57S Condensates Concentrate α/β-Tubulin Dimers for Microtubule Assembly.

**Fig. 4.**
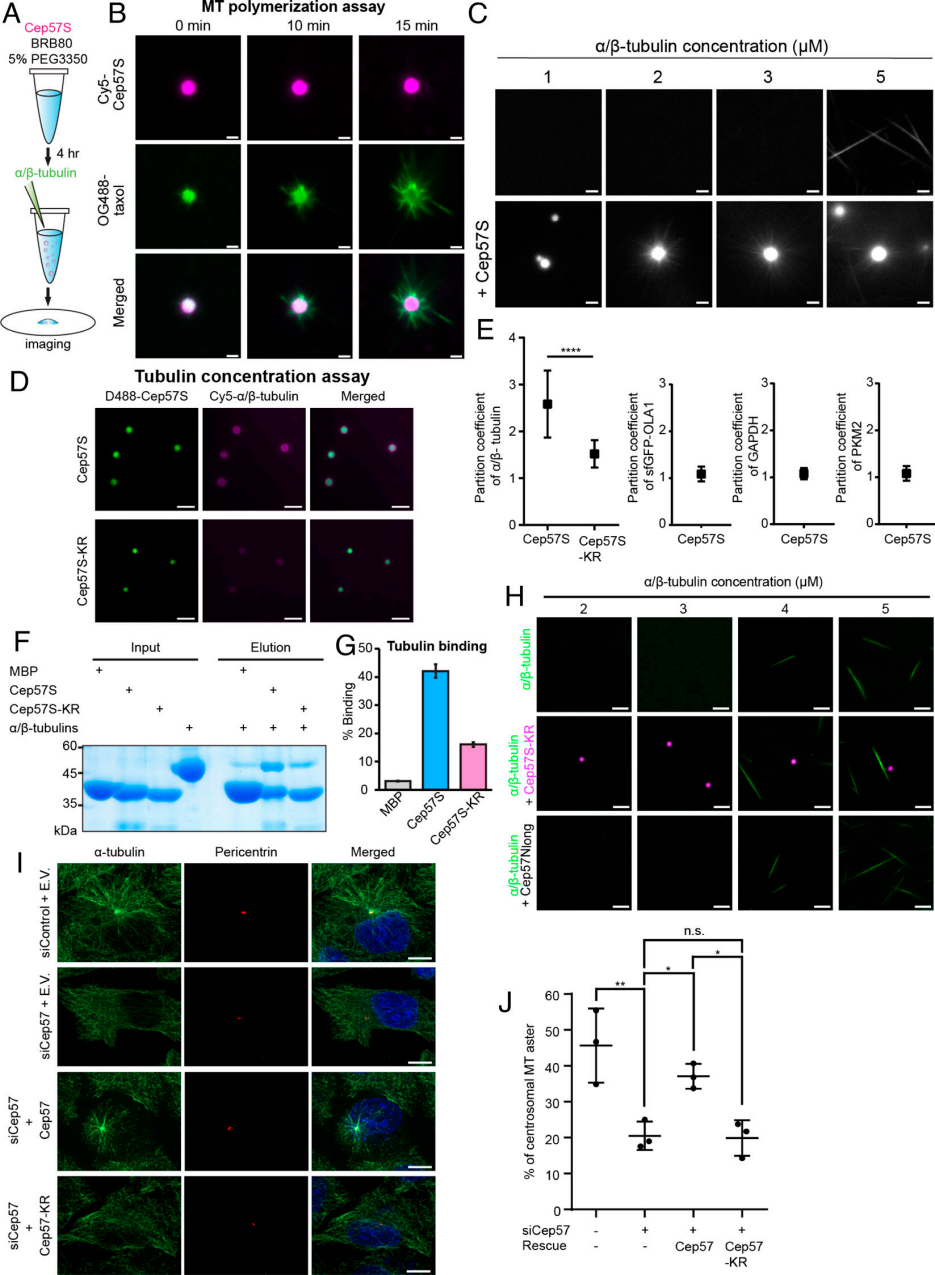
The Cep57 scaffold concentrates α/β-tubulin for microtubule assembly. (*A*) Schematic of α/β-tubulin polymerization or concentration assay by Cep57S condensates, formed by incubating 5 μM Cep57S at 22 °C supplemented with 5% PEG3350 for 4 h. (*B*) TIRF images of Cep57S taken at the indicated time points after adding tubulin. Cep57S was labeled with 5% Cy5. α/β-tubulin concentration, 2 μM. OG488-taxol marked polymerized microtubules. (Scale bar, 2 μm.) (*C*) Critical concentration measurements in the absence or presence of Cep57S. (*D*) D488-Cep57S or D488-Cep57S-KR condensates with Cy5-tubulin dimers in the presence of 66 μM nocodazole. Images were taken 20 min after the addition of tubulin. (Scale bar, 5 μm.) (*E*) Quantification of the α/β-tubulin partition coefficient to Cep57S (n = 69) or Cep57-KR (n = 76) condensates in experiments from panel (*D*) pooled from three repeats; and the partition coefficient of 10 μM sfGFP-OLA1 (n = 83), 5 μM FITC-GAPDH (n = 88), or 5 μM Cy3-PKM2 (n = 67) to Cep57S condensates pooled from three repeats. [****] *P* < 0.0001. Data represent mean ± SD. (*F*) His-tag pull-down assay of tubulin with His-Cep57S and His-Cep57S-KR. His-MBP was used as the negative control. (*G*) Quantification of binding of α/β-tubulin in experiments from figure (*F*). The percentage of binding was defined as the intensity of tubulin in elution divided by that in input. N = 3. (*H*) Critical concentration measurements of microtubule nucleation in the absence or presence of 5 μM Cep57S-KR or in the presence of 5 μM Cep57N_long_ (aa 56 to 264 including the K/R motif). (*I*) Control or Cep57-knockdown HeLa cells expressing the indicated constructs subjected to the microtubule regrowth analysis. Microtubule regrowth was carried out for 2 min after microtubule depolymerization by 1 h of cold treatment with 16.6 μM nocodazole. Green, α-tubulin; red, pericentrin; blue, DNA. (Scale bar, 10 μm.) (*J*) Percentage of cells with regrown centrosomal microtubule asters in experiments from panel (*I*). HeLa cells were sequentially transfected from *Left* to *Right* with (negative control siRNA + mCherry empty vector), Cep57 siRNA + mCherry empty vector), (Cep57 siRNA + Cep57-mCherry), and (Cep57 siRNA + Cep57-KR-mCherry). Each data point was derived from observations of 100 to 200 cells. [*] *P* < 0.05; [**] *P* < 0.01. N = 3.

TPX2 and tau condensates nucleate microtubule by concentrating tubulin dimers in reconstituted systems (24, 35, 36). Cep57 CTD alone binds to and bundles microtubules (27), and the depletion of Cep57 impairs spindle microtubule assembly in cells (13). These findings suggest that Cep57 condensates may have microtubule assembly activity. To test this idea, we performed a microtubule nucleation assay in BRB80 and 5% PEG3350, which prevents protein precipitation that formed upon mixing α/β-tubulin with preexisting Cep57S condensates. As illustrated in Fig. 4*A*, we performed the assays using OG488-taxol to mark polymerized microtubules. Microtubules emanated from Cep57S condensates immediately after adding α/β-tubulin (Fig. 4*B*). Over time, microtubule asters became more evident with a morphology resembling microtubule asters found in cells (Fig. 4*B*). To determine whether Cep57S condensates facilitate microtubule nucleation, we determined the critical tubulin concentration for spontaneous assembly, which was 5 μM in our assay system (Fig. 4*C*). Cep57S condensates further lowered the tubulin threshold to ~2 μM. Next, we examined the partition of tubulin dimers using Cy5-tubulin (10 μM, 5% labeled) in the presence of nocodazole, which inhibits tubulin polymerization. Cep57S condensates concentrated α/β-tubulin dimers by about 2.6-fold (Fig. 4 *D* and *E*), indicating that the tubulin concentration in condensates is above the critical concentration for microtubule assembly when the bulk tubulin concentration is 2 μM. In contrast, non-Cep57 interacting proteins sfGFP-OLA1 (78 kDa), GAPDH (144 kDa as a tetramer), and PKM2 (232 kDa as a tetramer) demonstrate a partition coefficient of ~1.1. Therefore, Cep57 condensate selectively recruits tubulin. In addition, our data reveal that the interstitial space within Cep57 condensates can accommodate molecules of at least 11 nm in size, as estimated from the crystal structure of PKM2.

Electrostatic interactions often mediate interactions between α/β-tubulin and microtubule-associated proteins (MAPs) between positively charged motifs on MAPs and negative-charged tubulin surface (37, 38). To examine whether tubulin binds to the K/R motif of Cep57, we performed a pull-down assay. His-tagged Cep57S coeluted with α/β-tubulin, whereas the K/R mutation largely diminished tubulin binding (Fig. 4 *F* and *G*). This mutational binding analysis identified the poly-K/R motif as a tubulin binding site on Cep57, in addition to the known C-terminal domain (aa 278 to 491) (27). Moreover, the poly-K/R motif is the dominant binding site for tubulin in Cep57S since the KR mutation reduced the binding by ~65%. We further tested whether tubulin binding–deficient Cep57S-KR condensates can nucleate microtubules. Compared to the tubulin alone control, Cep57S-KR did not affect microtubule assembly (Fig. 4*H*), and the KR mutation reduced the partition coefficient of tubulin (Fig. 4 *D* and *E*). To delineate if LLPS of Cep57S is required for microtubule nucleation, we conducted microtubule nucleation assays on an LLPS-incompetent fragment (Cep57N_long_) containing only the NTD and poly-K/R motif. This fragment demonstrated no effect on microtubule assembly (Fig. 4*H*).

Combined, our data demonstrate that Cep57S condensates nucleate microtubules and the K/R motif contributes to tubulin recruitment. Given the functional significance, we renamed this K/R motif the LLPS and microtubule nucleation (LMN) motif.

Next, we examined the effect of the LMN motif on the microtubule assembly in cells. We performed a microtubule regrowth assay on cells whose endogenous Cep57 was knocked down by siRNA (*SI Appendix*, Fig. S3*C*). Microtubule disassembly was achieved by cold treatment in the presence of nocodazole for 1 h. After nocodazole was washed away, cells were incubated in a warm medium to allow for microtubule reassembly for 2 min. Aster formation was abolished in Cep57-depleted cells (Fig. 4 *I* and *J*). The overexpression of a siRNA-resistant Cep57 construct fully rescued this phenotype, whereas the LMN mutant failed. Through the in vitro and cellular assays, we demonstrate that Cep57 plays a role in the formation of centrosomal microtubule asters in an LMN motif-dependent manner.

### Cep63 Regulates LLPS and MT Nucleation Activity of Cep57.

**Fig. 5.**
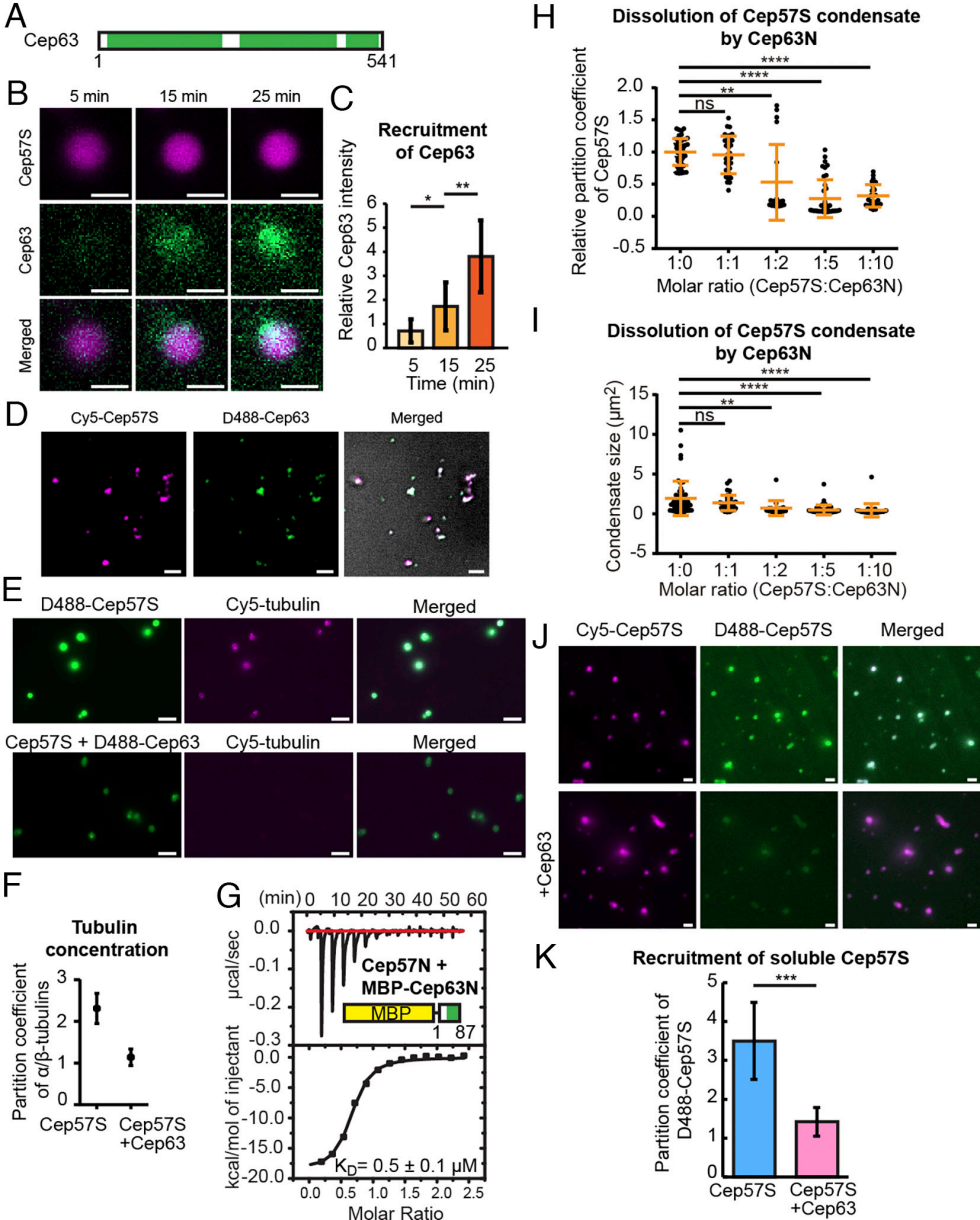
Cep63 negatively regulates Cep57S assembly and activity. (*A*) Domain architecture of Cep63 (Uniprot ID: Q96MT8-2). The predicted coiled-coil regions are green. (*B*) Time-lapse images of Cep63 recruited to Cep57S condensates. Condensates were formed by incubating Cep57S (13 μM, 5% Cy5 labeled) at 22 °C for 6 h. Image acquisition was started after the addition of Cep63 (6 μM, 5% D488 labeled). (Scale bar, 2 μm.) (*C*) Intensity of Cep63 in experiments from panel (*B*) (n = 8). (*D*) TIRF images of Cep57S-Cep63 cocondensates in a 10:1 ratio preincubated at 22 °C for 6 h prior to image acquisition. (Scale bar, 2 μm.) (*E*) Images of D488-Cep57S and Cep57S-D488-Cep63 droplets taken 20 min after the addition of 5 μM α/β-tubulin and 66 μM nocodazole. Condensates were formed by incubating 5 μM Cep57 with or without 5 μM Cep63 at 22 °C for 6 h. (Scale bar, 5 μm.) (*F*) Quantification of partition coefficient of α/β-tubulin in Cep57S or Cep57S-Cep63 droplets in experiments from panel (*E*). n = 51 (Cep57) and 102 (Cep57S-Cep63) pooled from three independent experimental repeats. (*G*) Interaction analysis of Cep57N to MBP-Cep63N by ITC. (*H* and *I*) Partition coefficient and size analysis of Cep57S condensates with increasing concentrations of MBP-Cep63N. The concentration of Cep57S was fixed at 2 μM. Data from two independent experiments were pooled for quantification; n = 51 (1:0), 34 (1:1), 19 (1:2), 39 (1:5), and 28 (1:10). (*J*) Images of Cy5-Cep57S or Cy5-Cep57S-Cep63 droplets with soluble D488-Cep57S. Condensates were formed by incubating Cep57S (10 μM, 5% Cy5 labeled) with 5% PEG3350 in low salt for 4 h at 22 °C. 5 μM of Cep63 was added and further incubated for 2 h. Finally, 0.4 μM of D488-Cep57S was added, followed by incubation for 2 more hours before image acquisition. For the control, the step of adding Cep63 was skipped. (Scale bar, 2 μm.) (*K*) Quantification of the D488-Cep57S partition coefficient in experiments from panel (*J*). n = 266 (Cep57S) and 139 (Cep57S + Cep63) pooled from three independent experimental repeats. [*] *P* < 0.05, [**] *P* < 0.01, [***] *P* < 0.001, and [****] *P* < 0.0001.

The LLPS of Cep57S is driven by multiple interactions among Cep57S molecules, which may provide sites for the regulation of LLPS, activity, and expansion from the existing assembly in the centrosome cycle. Previous studies revealed that N-terminal Cep57 interacts with Cep63, and this interaction is required for the PCM localization of Cep152 (28, 39). To study the role of Cep63 in Cep57 assembly, we purified full-length Cep63 to high purity (Fig. 5*A* and *SI Appendix*, Fig. S4*A*). The CD analysis of Cep63 showed it is highly helical as a coiled-coil protein should be (*SI Appendix*, Fig. S4*B*). In our LLPS system, we applied the soluble D488-labeled Cep63 to the preformed condensates and observed the gradual recruitment of Cep63 to the Cep57 scaffold over time (Fig. 5 *B* and *C*). When soluble Cep57S and Cep63 in high salt were simultaneously diluted in low salt to allow cocondensation, Cep63 exhibited a high partition coefficient of 10.7 ± 1.2 (n = 166) after 6 h (Fig. 5*D*). Interestingly, we noticed the shape of Cep57S-Cep63 cocondensates became amorphous, implying the material property of condensates was changed to a solid-like state (Fig. 5*D*).

Next, we investigated how Cep63 affects the recruitment of α/β-tubulin. In the experiments, we prepared condensates 4 h before supplementing 5 μM tubulin in the presence of nocodazole. Compared to the Cep57S control, Cep63 greatly reduced tubulin concentration within the cocondensates with a partition coefficient close to 1 (Fig. 5 *E* and *F*), indicating that soluble tubulin dimers could penetrate the cocondensates, but could not be locally enriched. How did Cep63 impede tubulin concentration by Cep57S? One possibility is that Cep63 blocks the tubulin-binding LMN motif on Cep57S. However, the reported Cep63 binding motif is distant from the LMN (28), making the blocking hypothesis less likely. The other possibility is that Cep63 reduces the density of LMN motifs in the condensate so that the tubulin concentration effect is diminished. If true, Cep57S’ partition coefficient will be lowered by Cep63. To test this idea, we used the N-terminal fragment of Cep63 (Cep63N) in the phase separation assays instead of the full-length protein, whose solubility was low. Cep63N has been reported to interact with Cep57 (28). To improve the solubility of Cep63N for biophysical measurements, we created a fusion of MBP and Cep63N. Our ITC data confirmed a strong interaction between Cep57N and MBP-Cep63N (K_D_ = 0.6 μM, Fig. 5*G*). Strikingly, MBP-Cep63N dissolved Cep57S condensates as the partition of Cep57S and the droplet size reduced considerably in a concentration-dependent manner (Fig. 5 *H* and *I* and *SI Appendix*, Fig. S4*C*). In the nanometer scale, SAXS measurements reveal the internal structural change induced by MBP-Cep63N. First, the Cep57S condensate exhibits a fractal network structure (*SI Appendix*, Fig. S5*A*). The slope in the fractal region became flattened as the amount of MBP-Cep63N increased (*SI Appendix*, Fig. S5*A*), indicating the reduction of the fractal network. The time course of dissolution is about 1 h from the time-dependent scattering analysis (*SI Appendix*, Fig. S5*B*). MBP-Cep63N reduced the fractal network of Cep57S condensates in a concentration- and time-dependent manner, correlating well with the microscopic observation (Fig. 5 *H* and *I* and *SI Appendix*, Fig. S4*C*). There is a possibility that MBP may contribute to the dissolution of Cep57S condensates. However, considering all the data together, it strongly indicates that Cep63 regulates the microtubule nucleation activity of Cep57S by the impediment of LLPS via disrupting the network structure of condensates.

In mitosis, the PCM expansion is critical for bipolar spindle assembly. This process is achieved by the accumulation of scaffolding proteins. Cep63 has been reported to play a negative role in PCM maturation. Downregulation of Cep63 protein level permits PCM expansion (6). Since Cep63N led to the dissolution of Cep57S condensates, we further tested whether Cep63 represses cocondensates to recruit soluble Cep57S in the bulk environment. In effect, preexisting condensates were marked by Cy5, and the supplemented Cep57S was marked by D488 at 0.4 μM below the critical concentration to keep D488-Cep57S soluble. Images were taken 2 h after supplementing soluble D488-Cep57S into the preexisting condensates. The Cep57S assembly concentrated soluble D488-Cep57S with a partition coefficient of ~3.5 (Fig. 5 *J* and *K*). In contrast, D488-Cep57S barely accumulated at the Cep63-Cep57S cocondensates with a partition coefficient of ~1.3. Our data demonstrate that Cep63 could intervene in the Cep57S assembly, thereby suppressing its LLPS expansion. Thus, modulating LLPS by client proteins is a means to control the structural expansion and functional activity of scaffolding proteins.

### Disruption of Cep57 Multivalent Interactions Resulted in PCM Disorganization, Centriole Disengagement, and Centrosome Amplification.

**Fig. 6.**
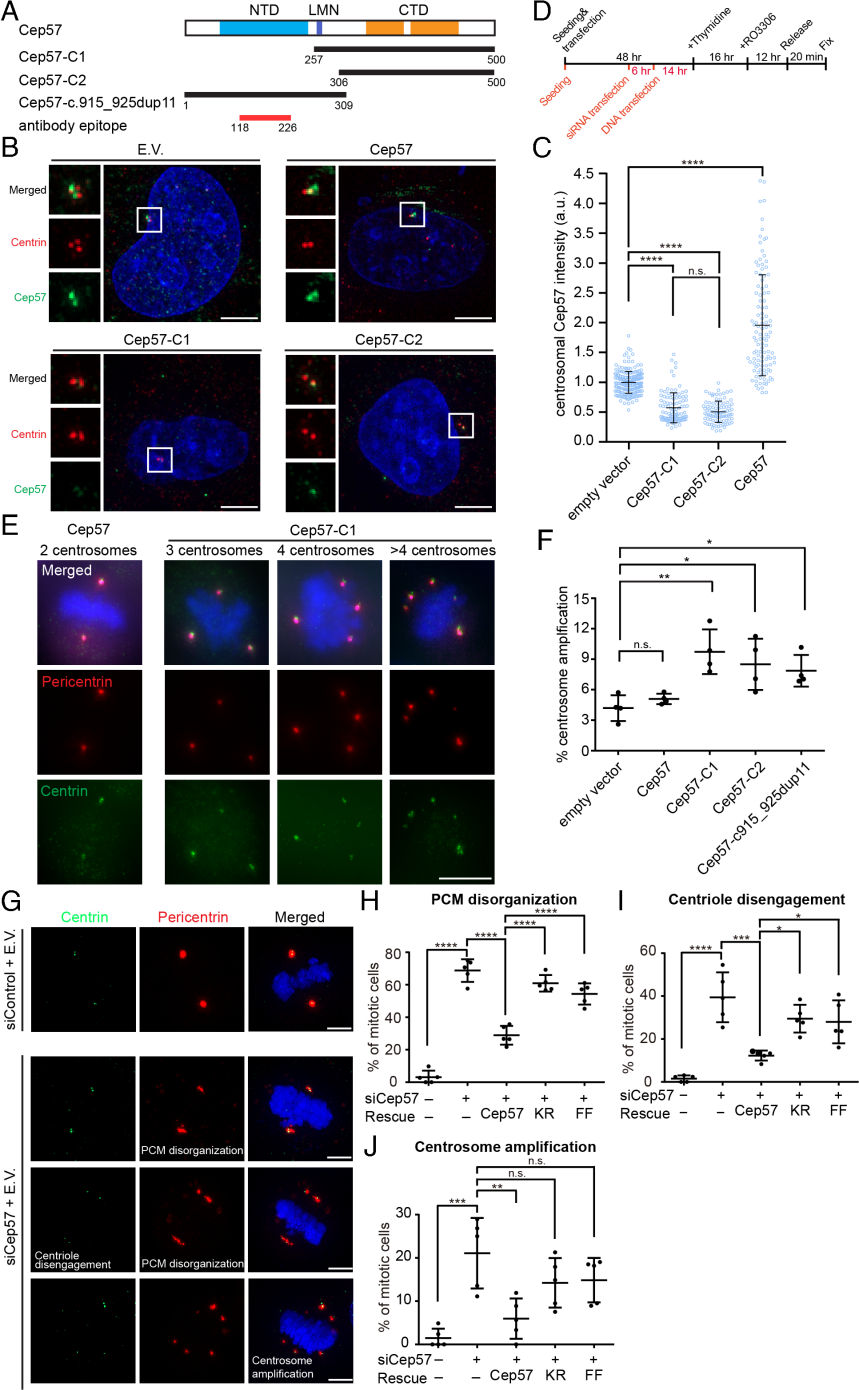
Multivalent interactions of Cep57 regulate the structure and number of human centrosomes. (*A*) Cep57 truncation constructs overexpressed in HeLa cells. (*B*) Fluorescence images of interphase HeLa cells overexpressing mCherry empty vector, Cep57-mCherry, Cep57-C1-mCherry, and Cep57-C2-mCherry for 16 to 18 h. Centrin, red; Cep57, green; DNA, blue. (Scale bar, 5 μm.) (*C*) Quantification of centrosomal Cep57 intensity of interphase HeLa cells overexpressing the indicated constructs from panel (*B*). n = 211, 112, 104, 92 pooled from three experiments. (*D*) Synchronization scheme for panels (*E*) (black) and (*G*) (red + black). (*E*) Fluorescence images of mitotic cells overexpressing Cep57 or Cep57-C1 for 48 h. Pericentrin, red; centrin, green; DNA, blue. (Scale bar, 10 μm.) (*F*) Quantification of centrosome amplification, defined as more than two centrosomes in a cell, from cells overexpressing the indicated constructs. Each data point was collected from 100 to 300 cells. N = 4. (*G*) Fluorescence images of mitotic HeLa cells sequentially transfected with negative control siRNA and mCherry empty vector, or Cep57 siRNA and mCherry empty vector. Centrin, green; pericentrin, red; DNA, blue. (Scale bar, 5 μm.) (*H*–*J*) Percentage of mitotic cells with indicated phenotypes. HeLa cells were sequentially transfected from *Left* to *Right* with (negative control siRNA + mCherry empty vector), (Cep57 siRNA + mCherry empty vector), (Cep57 siRNA + Cep57-mCherry), (Cep57 siRNA + Cep57-KR-mCherry), and (Cep57 siRNA + Cep57-FF-mCherry), followed by synchronization. Each data point was collected from 30 to 50 mitotic cells. N = 5. [*] *P* < 0.05, [**] *P* < 0.01, [***] *P* < 0.001, and [****] *P* < 0.0001.

Our in vitro study shows that the NTD, CTD, and LMN motif are crucial for promoting phase separation of Cep57. To investigate the significance of these multivalent interactions in the centrosomal recruitment of Cep57, we created two truncation mutations Cep57-C1 and Cep57-C2 (Fig. 6*A*). We used an antibody that recognizes the N-terminal region of Cep57 (residues 118 to 226) for immunofluorescence imaging assays, but not the Cep57-C1 and Cep57-C2 constructs. The expression of truncation constructs did not affect the endogenous Cep57 level as compared to the vector control (*SI Appendix*, Fig. S6*A*). However, the amount of endogenous Cep57 at centrosomes decreased, while the full-length construct increased the centrosomal Cep57 level (Fig. 6 *B* and *C*). Our data indicate that truncation mutants, which compete for the multivalent interactions of Cep57, inhibit LLPS in vitro and centrosomal recruitment in cells.

Furthermore, we investigated whether the truncation constructs have effects in centrosome duplication. HeLa cells expressing these constructs were synchronized to the M phase by thymidine and subsequent RO-3306 treatment (Fig. 6*D*). Cells were subjected to immunostaining 20 min after release from the G2 block. Overexpression of the empty vector control or full-length Cep57 resulted in a similar degree of centrosome amplification (Fig. 6 *E* and *F*). In contrast, overexpression of Cep57-C1, Cep57-C2, or Cep57-c.915_925dup11 [C-terminal deletion mutation found in mosaic-variegated aneuploidy (MVA) patients (29–31)] induced centrosome amplification.

Cep57 depletion causes centrosome amplification through precocious centriole disengagement in mitosis, which licenses new centrosome formation (40). This finding prompted us to investigate the role of Cep57 multivalent interactions in centriole engagement. We performed siRNA experiments against Cep57, which resulted in PCM disorganization (amorphous shape or fragmentation of PCM), centriole disengagement (lengthening the distance between the centriole pair), and centrosome amplification (more than two centrosomes in a cell) during mitosis (Fig. 6 *G–J*). Introducing the siRNA-resistant Cep57 construct can restore its function. In contrast, siRNA-resistant Cep57-KR and Cep57-FF showed comparable expression levels (*SI Appendix*, Fig. S6*B*) to Cep57 but failed to rescue these phenotypes to the same degree as the wild type (Fig. 6 *G–J*). The KR and FF mutations impair the LLPS of Cep57S in vitro and induce premature centriole disengagement during mitosis. We conclude that Cep57 maintains the PCM integrity through multivalent interactions.

Numerical and structural centrosome aberration can cause enhanced cell invasion (41, 42). To investigate the role of Cep57 LLPS in cell motility, we assessed the migration ability of U2OS cells expressing these Cep57 constructs in Boyden chambers. Although these constructs have minimal effect on cell migration, we still observed that Cep57-C1 promoted U2OS migration compared to the full length and control (*SI Appendix*, Fig. S6 *C* and *D*). Together, these cell assays demonstrated the biological significance of Cep57 multivalent interactions in centrosome integrity.

## Discussion

### Phase Transition of Cep57 and Its Implication for PCM Formation.

**Fig. 7.**
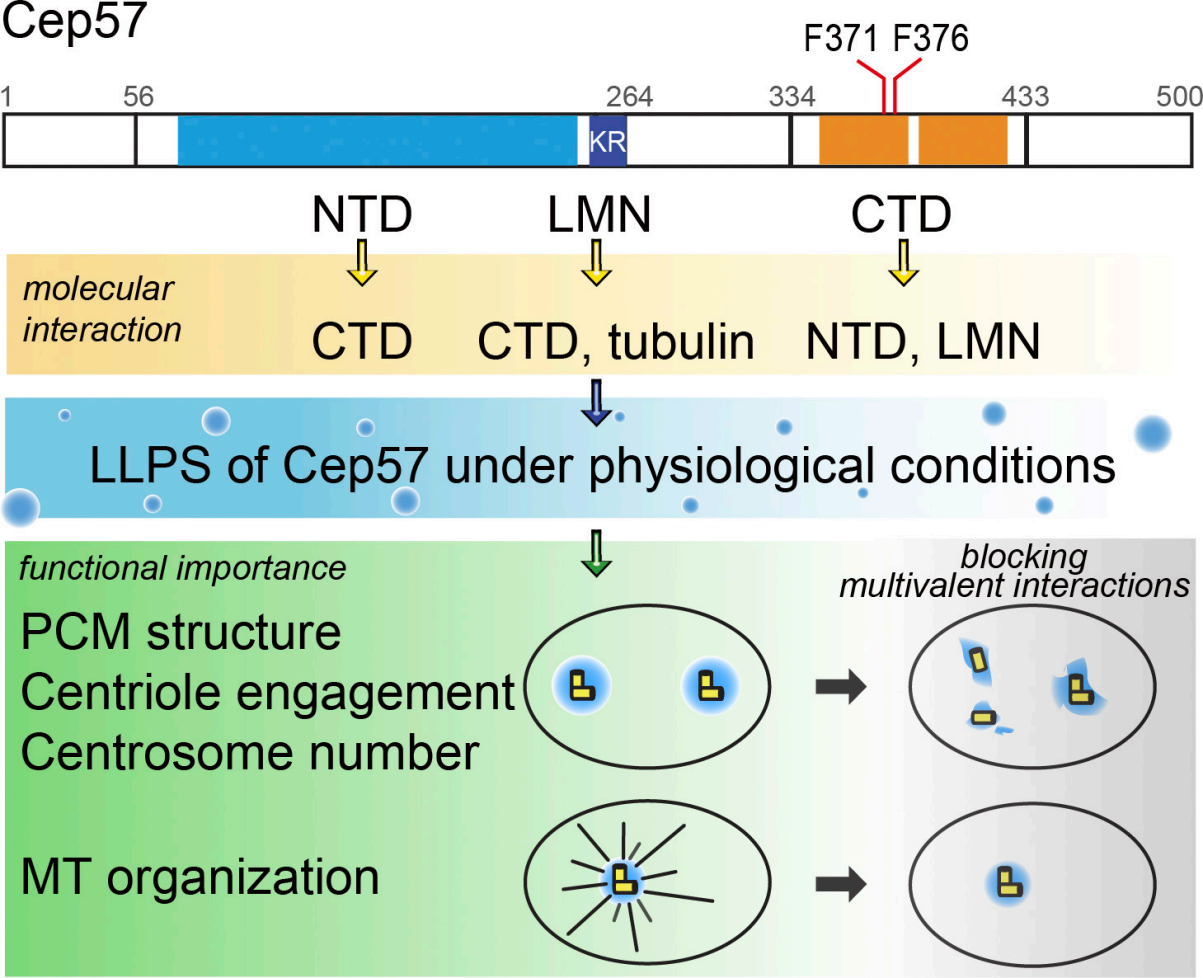
A model for the assembly of human Cep57 in centrosome regulation. Domain organization of Cep57 is depicted with interactions that drive LLPS under physiological conditions. SiRNA rescue experiments reveal that the multivalent interactions of Cep57 are essential for PCM organization, and the LMN motif facilitates microtubule aster formation.

Our work elucidates how human PCM protein Cep57 assembles into micron-sized biomolecular condensates through liquid–liquid phase separation by employing various tools in vitro. Both purified Cep57 and Cep57S undergo LLPS, driven by at least three interacting regions, including NTD, LMN motif, and CTD (Fig. 7). The critical concentration for LLPS of purified Cep57 is around 4 nM, similar to the cellular concentration of Cep57 (32). Cellular analyses support that the multivalent interactions of Cep57 maintain the structural organization of the PCM. Two LLPS-incompetent mutants (KR and FF) result in centriole disengagement, PCM disorganization, and centrosome amplification in Cep57-depleted cells (Figs. 6 *G*–*J* and 7). It demonstrates the importance of Cep57 assembly for the integrity of human centrosomes.

Pericentrin, a critical PCM scaffold for mitotic centrosome maturation, may utilize its conserved coiled-coil and low-complexity region for phase separation in cells (43). *Caenorhabditis elegans* SPD-5, *Drosophila* CNN, and human CDK5RAP2 are functional homologs with very low sequence similarity. Interestingly, the principles governing the self-assembly of SPD-5 and CNN scaffold are extremely similar. Both use two coiled-coil domains to promote intra- and intermolecular interactions for scaffold formation (23, 44). Similarly, Cep57 undergoes LLPS through the interaction between the dimeric NTD and CTD. These scaffolding proteins utilize minimally two dimeric/oligomeric coiled-coil domains to engage intra- and intermolecularly for PCM assembly. Upon this core principle, extra motifs such as the LMN and posttranslational modifications such as phosphorylation could further enhance this assembly process with high specificity. It has been shown that phosphorylation and dephosphorylation control the CNN and SPD-5 scaffold (24, 44, 45). As coiled-coil domains often provide binding sites for other proteins, the coiled-coil mediated assembly provides a highly regulatory nature and can also function as a structural and signal hub. Here, our data suggest that Cep63 negatively controls the Cep57 assembly in a concentration-dependent manner. Cells may regulate the dynamic structural assembly of centrosomes by fine-tuning the ratio of Cep57 and Cep63. More fascinating molecular interplays between centrosomal scaffold proteins await to be discovered.

### Functional Implications of Multivalent Interactions-Mediated PCM Assembly on Centrosome Functions.

We identified a tubulin-binding LMN motif in Cep57. This motif contributes to LLPS and facilitates microtubule assembly (Figs. 2 *F–J* and 4). In the presence of OG488-taxol and 5% PEG3350, the critical concentration for microtubule assembly is 4 to 5 μM. The addition of Cep57S condensates lowered this critical concentration to 2 μM, while the partition coefficient of tubulin is 2.6. These findings suggest that tubulin dimers within Cep57S droplets reach a concentration (around 5.2 μM) akin to the critical threshold for spontaneous microtubule assembly. It strongly argues that Cep57 promotes tubulin assembly by locally increasing tubulin dimer concentrations above the threshold required for spontaneous microtubule assembly. Importantly, this biochemical activity observed in the reconstitution system is mirrored in cellular contexts. The LMN motif is essential for microtubule aster formation, as observed in the microtubule regrowth assay (Fig. 4 *I* and *J*).

Cell cycle transition from the G2 to M phase is accompanied by PCM expansion, characterized by the accumulation of scaffold proteins in centrosomes to boost microtubule organizing activity for bipolar spindle formation (18). Our work finds that Cep63 limits the assembly of the Cep57S scaffold. At high molar ratios, Cep63N dissolved Cep57S condensate. The fusion of an MBP tag to Cep63N increases the solubility of this construct, raising the possibility that MBP may also play a role in dissolving the droplets. Moreover, Cep63 altered the material property of Cep57S condensates from a liquid-like to a more solid-like state, as the morphology of the Cep57S-Cep63 condensates changed to amorphous (Fig. 5*D*). It was reported that the percolated network of SPD-5 failed to facilitate microtubule formation (24). Here, we showed that Cep63 inhibited tubulin concentration by Cep57S condensate and suppressed the self-expansion of the condensate (Fig. 5). Biochemically, LLPS confers Cep57 microtubule nucleation activity by concentrating tubulin dimers via the LMN motif. Cep63 constrains Cep57’s activity, likely by reducing the LLPS of Cep57. Thus, compositional control of the centrosome, such as changing the ratio of Cep57 and Cep63, might be a mechanism the cell uses to remodel microtubule organization globally.

In addition to the dominant γ-TuRC templated mechanism, recent evidence showed that liquid–liquid phase separation grants tubulin-associated proteins activity for microtubule nucleation. Such cases include TPX-2, BugZ, Tau, and SPD-5/TPXL-1/ZYG-9 condensates (24, 35, 36, 46). Assisted by TPXL-1 and ZYG-9, SPD-5 cocondensate can concentrate tubulin to promote microtubule aster formation (24). Analogous to TPX-2, BugZ, and Tau, our data demonstrate that the Cep57 scaffold can locally enrich α/β-tubulin dimers and catalyze microtubule nucleation. These findings suggest that multivalent interactions-mediated assembly of PCM scaffold proteins can directly support the function of the mitotic centrosome, in addition to organizing the centrosome architecture and recruiting microtubule-organizing factors. It seems that not only the assembly mechanism but the LLPS-mediated functionality of PCM scaffold proteins are evolutionarily similar from worms to humans despite low sequence similarity. Ultimately, the synergy between these microtubule nucleating PCM scaffolds and γ-TuRC makes a robust mitotic centrosome for faithful cell division.

### Self-Assembly and Human Diseases Associated with Mutations of Cep57.

Cep57 mutations are genetically linked to mosaic-variegated aneuploidy (MVA), a rare disease characterized by an abnormal number of chromosomes. The MVA syndrome manifests in various disorders, including skeletal anomalies, microcephaly, and childhood cancers (29, 31, 47, 48). The disease mutations on Cep57 all lead to protein truncations lacking the C-terminal coiled-coil domain (c241c>t; c.520_521delGA; c.915_925dup11) (30, 31). Cellular studies from patient-derived cells revealed that Cep57 disease mutation leads to centrosome amplification (5, 29).

Of the highest occurrence, the Cep57 disease mutation c.915_925dup11 promoted centrosome abnormalities in MVA patient-derived cells, and homozygous *Cep57 *^(c.915_925dup11/c.915_925dup11)^ and heterozygous *Cep57 *^(+/c.915_925dup11)^ mouse MEF cells (29). Moreover, the heterozygous mutation enhanced cancer incidence in the mouse model (29). In our cell assays, overexpression of this disease construct resulted in centrosome amplification. Based on our biochemical analyses, this construct alone cannot phase separate but can inhibit the LLPS of Cep57. Our work provides a molecular mechanism for Cep57 mutation-mediated MVA syndrome. Truncation mutations in the Cep57 scaffold disrupt the integrity of the centrosome by interfering with its multivalent interaction network. Our findings suggest LLPS is essential in human centrosomes, and crippling LLPS by mutations leads to centrosome aberration, manifested as a human genetic disease. Primary microcephaly is a genetic disorder caused by mutations in a collection of proteins involved in DNA repair and centrosome function. However, molecular mechanisms for microcephaly mutations in centrosomal proteins, such as Cep135, Cep152, and WDR62, remain clouded (49). Interestingly, the disease mutations identified in these genes all resulted in protein truncation (50–52). It is worth investigating whether these proteins assemble by multivalent interactions and, if so, what is the effect of the truncation. Our study on human PCM scaffold proteins provides insight for a better understanding of centrosome-associated diseases at the molecular level and paves the road toward disease treatment.

## Materials and Methods

Key reagents and resources are in *SI Appendix*, Table S3.

### Cloning and Protein Purification.

All the constructs were cloned from the human cDNA library into a pCool2-His, pmCherry-N1, pmEGFP, or HA vector plasmid. Cep57 and Cep63 constructs were overexpressed by *Escherichia coli* Rosetta 2, which were lysed by Avestin Emulsflex C3 homogenizer at 4 °C. Clarified lysate was purified by affinity chromatography (Ni-NTA agarose, Qiagen), followed by gel filtration chromatography (Superdex 200, Cytiva) in 1 M NaCl. SF9 cells overexpressing sfGFP-Cep57 were disrupted by Dounce homogenization. Clarified lysate was purified by tandem affinity purification for 6× His tag and twin-strep-tag (Strep-Tactin Sepharose resin, IBA Lifesciences). Purified proteins were concentrated, flash-frozen by liquid nitrogen, and stored at −80 °C. Protein concentrations were determined by UV absorbance at 280 nm.

### Isothermal Titration Calorimetry.

The polybasic LMN peptide was synthesized by Kelowna International Scientific Inc., and dissolved in ITC buffer [150 mM potassium phosphate pH 7.5, 1 mM 2-mercaptoethanol, and 10% glycerol (w/v)]. All samples were dialyzed against the ITC buffer. The measurements were conducted at 25 °C or 15 °C as listed in *SI Appendix*, Table S1 (MicroCal iTC200, GE Healthcare). Curve fitting was accomplished in Origin**®** according to a single binding site model.

### His-Tag Pull-Down Assay.

Cep57S were incubated in a Ni-NTA spin column (Qiagen) for 30 min, followed by three cycles of rebinding at 4 °C, and washed three times by binding buffer [1X PBS pH 8.0, 1 mM 2-mercaptoethanol, 20 mM imidazole, 0.1% Triton X100, and 10% glycerol (w/v)]. 0.1 mg of α/β-tubulin (Cytoskeleton, Inc.) was then incubated in the column for 30 min, followed by three cycles of rebinding at 4 °C. After five washes, proteins were eluted using the binding buffer with 400 mM imidazole and subjected to SDS-PAGE analysis.

### Condensate and Microtubule Assembly Assays.

Condensate assembly was conducted by diluting concentrated samples in the assay buffer [20 mM Tris-HCl pH 8.0, 1 mM 2-mercaptoethanol, 1 mM PMSF, and 10% glycerol (w/v)]. For microtubule assembly assays, Cep57S condensates were prepared in BRB80 buffer supplemented with 150 mM NaCl, 10% glycerol, and 5% PEG3350 at 22 °C. After 4 h of incubation, Cep57S condensates were supplemented with 1 mM Mg/GTP, 0.5 μM OG-488 taxol (Thermo Fisher Scientific), and α/β-tubulin (Cytoskeleton, Inc.). For the tubulin concentration assay, 66 μM nocodazole (Selleckchem) was added to inhibit microtubule nucleation before supplementing 5 μM Cy5-tubulins (5% labeled). Each assay was repeated three times. The size and intensity of the condensates were quantified by applying “image threshold” and “particle analysis” functions on FIJI (53). All assembly assays were repeated three times.

### FRAP Analysis.

The FRAP measurements were conducted by “photobleaching” and “time series” functions of ZEN blue 3.1 on ZEISS LSM 800, using a 63× oil objective. The degree of photobleaching was set to 50%. After photobleaching, the images were taken every 15 s for young droplets and 30 s for old droplets. Data fitting was performed by Prism8 using a one-phase association model [% recovery = Plateau * (1 – e^−Time/Tau^)].

### SEC-MALS.

SEC-MALS experiments were performed by using Superdex 200 Increase 5/150 GL (Cytiva) equipped with three detectors: an Agilent 1260 DAD (UV), a Wyatt DAWN-HELEOS-II detector (MALS), and a Wyatt Optilab T-Rex differential refractive index detector (RI) with a buffer containing 20 mM HEPES pH7.5, 150 mM NaCl, and 1 mM DTT. Data were analyzed by Wyatt Astra software (version 7.3.1).

### Size Exclusion Chromatography Coupled to Small- and Wide-Angle X-ray Scattering (SEC-SWAXS).

SEC-SWAXS experiments were performed at the TPS 13A BioSWAXS beamline of the National Synchrotron Radiation Research Center, Taiwan (54, 55) in a buffer containing 20 mM Tris-HCl pH 8.0, 150 mM/1 M NaCl, and 1 mM 2-mercaptoethanol. The frame data of well-overlapped SAXS profiles were averaged and subtracted with buffer scattering using the TPS 13A SWAXS Data Reduction Kit (Ver. 3.6), and analyzed using ATSAS 3.1.3 (56).

### Protein Crystallography.

The His-Cep57C crystal was grown in a screen containing 0.1 M Sodium chloride, 0.1 M HEPES pH 7.5, and 1.6 M Ammonium sulfate at 22 °C by the hanging drop vapor diffusion method. Diffraction data were collected at 100 K at beamline TLS 13B, NSRRC, Taiwan. Data were processed by HKL2000 (57). The crystal structure of Cep57C was solved by the molecular replacement program Phaser-MR in Phenix (58) using a previously determined structure (PDB ID: 4L0R) as the search model. Structural refinement and model building were carried out using Phenix (58) and Coot (59) iteratively. The structure display was created by PyMOL (Schrodinger).

### Cell Assays.

Transient transfections of siRNA (MDBio, Inc.) and plasmid DNA were performed using TransIT-X2 (Mirus Bio) or FuGENE®HD (Promega). For the microtubule regrowth assay, cells were cold-treated with nocodazole, followed by microtubule reassembly. Primary antibodies are listed in *SI Appendix*, Table S3.

### Quantification and Statistical Analysis.

Statistical analyses for this study were conducted using Prism (GraphPad). Two-tailed student’s *t* test was used to compare two groups, while one-way ANOVA along with Dunnett’s multiple comparison test was used for comparing more than two groups. The data were presented as mean ± SD, and the *P*-values were indicated in the figure legends.

## Supplementary Material

Appendix 01 (PDF)

## Data Availability

The coordinates and structure factors for the crystal structure of Cep57C were deposited to the protein databank under the accession number: 8IBH (60).
