# Supplementary material for: Cep57 regulates human centrosomes through multivalent interactions
Source: Proc Natl Acad Sci U S A. 2024 Jun 10;121(25):e2305260121. doi: 10.1073/pnas.2305260121 (PMC11194501; doi:10.1073/pnas.2305260121)
Supplement: Supplementary file 1 — Appendix 01 (PDF) [file pnas.2305260121.sapp.pdf]

Supporting Information

Cep57 Regulates Human Centrosomes through Multivalent Interactions

Hung-Wei Yeh[1], Po-Pang Chen[1], Tzu-Chen Yeh[1], Shiou-Lan Lin[1], Yue-Ting Chen[1], Wan-Ping Lin[1], Ting Chen[1], Jia Meng Pang[2], Kai-Ti Lin[2], Lily Hui-Ching Wang[3], Yu-Chun Lin[4], Orion Shih[5], U-Ser Jeng[5,6], Kuo-Chiang Hsia[7], Hui-Chun Cheng[1*]

[1]Institute of Bioinformatics and Structural Biology, National Tsing Hua University, Hsinchu 30013, Taiwan,
[2]Institute of Biotechnology, National Tsing Hua University, Hsinchu 30013, Taiwan,
[3]Institute of Molecular and Cellular Biology, National Tsing Hua University, Hsinchu 30013, Taiwan,
[4]Institute of Molecular Medicine, National Tsing Hua University, Hsinchu 30013, Taiwan,
[5]National Synchrotron Radiation Research Center, Hsinchu 30076, Taiwan,
[6]Department of Chemical Engineering, National Tsing Hua University, Hsinchu 30013, Taiwan
[7]Institute of Molecular Biology, Academia Sinica, Taipei 11529, Taiwan
*Corresponding Author: Hui-Chun Cheng (hccheng@life.nthu.edu.tw)

This PDF file includes:

Tables S1-S3

Figures S1-S6

Supplementary Materials and Methods

Table S1. ITC fitting results*

Ligand	Receptor	K_D (μM)	N value	ΔH (cal/mol)	ΔS (cal/mol/deg)	T (°C)
Cep57C	Cep57N	9.1 ± 1.2	0.7 ± 0.1	$-5.9 * 10^4$	-175.0	25
MBP-Cep63N	Cep57N	0.5 ± 0.1	0.6 ± 0.0	$-1.9 * 10^4$	-34.0	25
Cep57C	K/R peptide	46.7 ± 9.2	1.1 ± 0.2	$-4.2 * 10^3$	5.4	15

*A one-site binding model was used for fitting the data.

Table S2. Data collection and refinement statistics

	Cep57C (PDB: 8IBH)
**Data collection**	
Space group	P 64 2 2
Cell dimensions	
a, *b*, *c* (Å)	62.94 62.94 81.49
α, β, γ (°)	90 90 120
Resolution (Å)	30.00-1.98 (2.05-1.98)
R_{merge}	0.058 (0.90)
$I / \sigma I$	38.1 (2.0)
Completeness (%)	99.3 (100.0)
Redundancy	9.2 (9.7)
**Refinement**	
Resolution (Å)	2.10
No. reflections	5441/ 272
R_{work} / R_{free} (%)	23.8 / 25.7
No. atoms	616
Protein	603
Water	13
B-factors	45.97
Protein	46.08
Water	40.82
R.m.s. deviations	
Bond lengths (Å)	0.016
Bond angles (°)	1.30
Ramachandran favored (%)	100

*Values in parentheses are for highest-resolution shell.

Table S3. Key resources table

REAGENT or RESOURCE	SOURCE	IDENTIFIER
**Antibody**		
Anti-Centrin Antibody (20H5)	MERCK	Cat# 04-1624; RRID: AB_10563501
Anti-Pericentrin Antibody (EPR21987)	Abcam	Cat# ab220784
Anti-Cep57 Antibody	ThermoFisher (IF and western blot)	Cat# PA5-53777; RRID: AB_2639689
Anti-Cep57 Antibody	GeneTex (western blot of overexpressed Cep57, Cep57KR, Cep57FF)	Cat# GTX115931; RRID:AB_10622106
Anti-α-tubulin Antibody (B-5-1-2)	Santa Cruz	Cat# Sc-23948; RRID: AB_628410
Anti-HA Antibody (F-7)	Santa Cruz	Cat# Sc-7392; RRID: AB_627809
Alexa Fluro 647-conjugated donkey anti-mouse IgG	ThermoFisher	Cat# A31571; RRID: AB_162542
Alexa Fluro 488-conjugated goat anti-mouse IgG	Invitrogen	Cat# A11001; RRID: AB_2534069
Alexa Fluro 594-conjugated donkey anti-rabbit IgG	Invitrogen	Cat# A21207; RRID: AB_141637
Anti-GFP Antibody (B-2)	Santa Cruz	Cat# Sc-9996; RRID: AB_627695
**Chemicals**		
Nocodazole	Selleckchem	Cat# R17934
Oregon Green 488-taxol	ThermoFisher	Cat# T34075
Western Lightning ECL Pro, Enhanced Chemiluminescent Substrate	Perkin Elmer	Cat# NEL121001EA
Coomassie brilliant	BIO-RAD	Cat# 161-0406

blue G-250		
Poly-L-lysine solution	Sigma-Aldrich	Cat# P8920
Thymidine	Sigma-Aldrich	Cat# 50-89-5
PIPES	Sigma-Aldrich	Cat# P1851
RO3306	Sigma-Aldrich	Cat# 872573-93-8
DAPI	Invitrogen	Cat# D1306
TurboFect™ Transfection Reagent	ThermoFisher	Cat# R0531
DMEM/High glucose with L-glutamine, sodium pyruvate	Cytiva	Cat# SH30243.01
FuGENE®HD	Promega	Cat# E2311
Opti-MEM™ I Reduced Serum Medium	Gibco	Cat# 31985070
K/R peptide	Kelowna	N/A
α/β-tubulins (porcine brain)	Cytoskeleton, Inc	Cat# T240-B
α/β-tubulins (HiLyte647™ dye labeled; porcine)	Cytoskeleton, Inc	Cat# TL670M-A
Dylight 488	ThermoFisher	Cat# 46402
Cy®5 Mono NHS Ester	Cytiva	Cat# PA15101
**Culture Dish**		
Cell culture dish (90 x 20 mm)	SPL life science	Cat# 20101
8-well chambered coverglass	ThermoFisher	Cat# 155411
Cell culture dish (3.5 mm)	Alpha plus	Cat# 16235-1S15
**Resins and columns**		
Ni-NTA	Qiagen	Cat# 30230
Ni-NTA spin column	Qiagen	Cat# 31314
Superdex 200 Increase 10/300 GL	Cytiva	Cat# GE17-5175-01
Strep-Tactin Sepharose resin	IBA Lifesciences	

**Recombinant DNA**		
mCherry-Cep57	This paper	N/A
SfGFP-Cep57	This paper	N/A
Cep57S	This paper	N/A
Cep57tevS	This paper	N/A
Cep57S-KR	This paper	N/A
Cep57S-FF	This paper	N/A
Cep57S-CM2L	This paper	N/A
Cep57N	This paper	N/A
Cep57N-KR	This paper	N/A
Cep57C	This paper	N/A
Cep57C-FF	This paper	N/A
Cep63	This paper	N/A
MBP-Cep63N	This paper	N/A
Cep57	This paper	N/A
Cep57-C1	This paper	N/A
Cep57-C2	This paper	N/A
Cep57-C.915_925dup11	This paper	N/A
**siRNA sequence**		
siRNA control	5'- UUCUCCGAACGUGUCACGUTT-3'<br>3'-TTAAGAGGCUUGCACAGUGCA-5'	
siRNA Cep57	5'- GGACCACAAAUAAGAAAGATT-3'<br>3'-TTCCUGGUGUUUAUUCUUUCU-5'	
**Deposited data**		
Crystal structure of Cep57C	Protein Data Bank	PDB: 8IBH
**Software and algorithms**		
GraphPad Prism 6	GraphPad	https://www.graphpad.com/<br>RRID:SCR_002798
HKL2000	Z. Otwinowski and W. Minor, 1997	https://hkl-xray.com/hkl-2000<br>RRID:SCR_015547
FIJI	Schindelin et al., 2012	https://imagej.net/Fiji<br>RRID:SCR_002285
PyMol	WL, Delano, 2002	https://pymol.org/2/<br>RRID:SCR_000305
Phenix	Adams et al, 2002	https://phenix-online.org/

		RRID:SCR_014224
Coot	P. Emsley and K. Cowtan, 2004	https://www2.mrc-lmb.cam.ac.uk/personal/pemsley/coot/ RRID:SCR_014222
Jalview	Waterhouse et al, 2009	https://www.jalview.org/ RRID:SCR_006459
Clustal X	Larkin et al, 2007	http://www.clustal.org/ RRID:SCR_017055

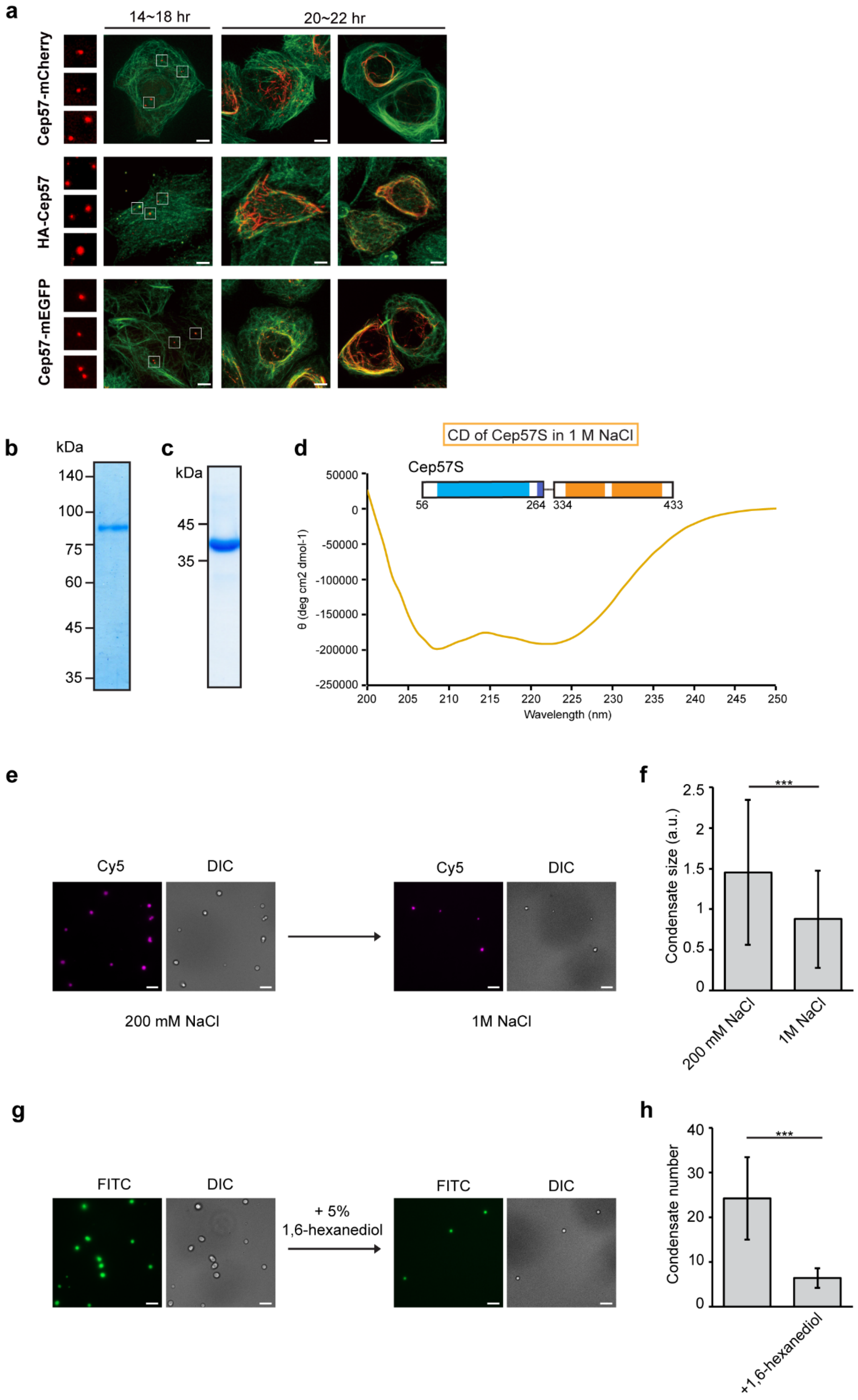
a
14~18 hr
20~22 hr
Cep57-mCherry
HA-Cep57
Cep57-mEGFP
b
kDa
140
100
75
60
45
35
c
kDa
45
35
d
CD of Cep57S in 1 M NaCl
Cep57S
56
264
334
433
θ (deg cm2 dmol-1)
Wavelength (nm)
e
Cy5
DIC
200 mM NaCl
1M NaCl
f
***
Condensate size (a.u.)
200 mM NaCl
1M NaCl
g
FITC
DIC
+ 5% 1,6-hexanediol
h
***
Condensate number
+1,6-hexanediol

Figure S1. Cep57S exhibited better protein quality than Cep57 and formed spherical condensates reversibly. a Fluorescence images of Cep57-mCherry, Cep57-mEGFP, and HA-Cep57 with indicated expression time in HeLa cells. Green, α-tubulin; red, Cep57. Scale bar, 5 μm. **b** SDS-PAGE profile of sfGFP-Cep57 visualized by Coomassie blue stain. **c** SDS-PAGE profile and **d** the circular dichroism spectra of purified Cep57S in buffer containing 20 mM Tris-HCl pH 8.0, 1 M NaCl, 1 mM 2-mercaptoethanol, 1 mM PMSF, and 10% glycerol (w/v). 10 μM of Cep57S was scanned in far-UV range (200-250 nm). **e** TIRF images showing Cep57S condensates dissolved by increasing salt concentration. Protein concentration was fixed at 12 μM. 5% of Cy5-Cep57S (magenta) was included. Scale bar, 5 μm. **f** Size of Cep57S condensates determined by FIJI in experiments from panel e; n = 36 (200 mM NaCl) and 58 (1 M NaCl) pooled from 3 technical repeats. Data represent mean ± SD. Statistical differences were assessed by two-tailed student's *t*-test; [***] p<0.001. **g** TIRF images showing Cep57S condensates dissolved by 1,6-hexandiol. Protein concentration was fixed at 12 μM. 5% of Dylight-488-Cep57S (green) was included. Scale bar, 5 μm. **h** Averaged number of condensates per image from 17 (without 1,6-hexanediol) and 13 (with 1,6-hexanediol) images pooled from 3 technical repeats. Data represent mean ± SD. Statistical differences were assessed by two-tailed student's *t*-test; [***] p<0.001.

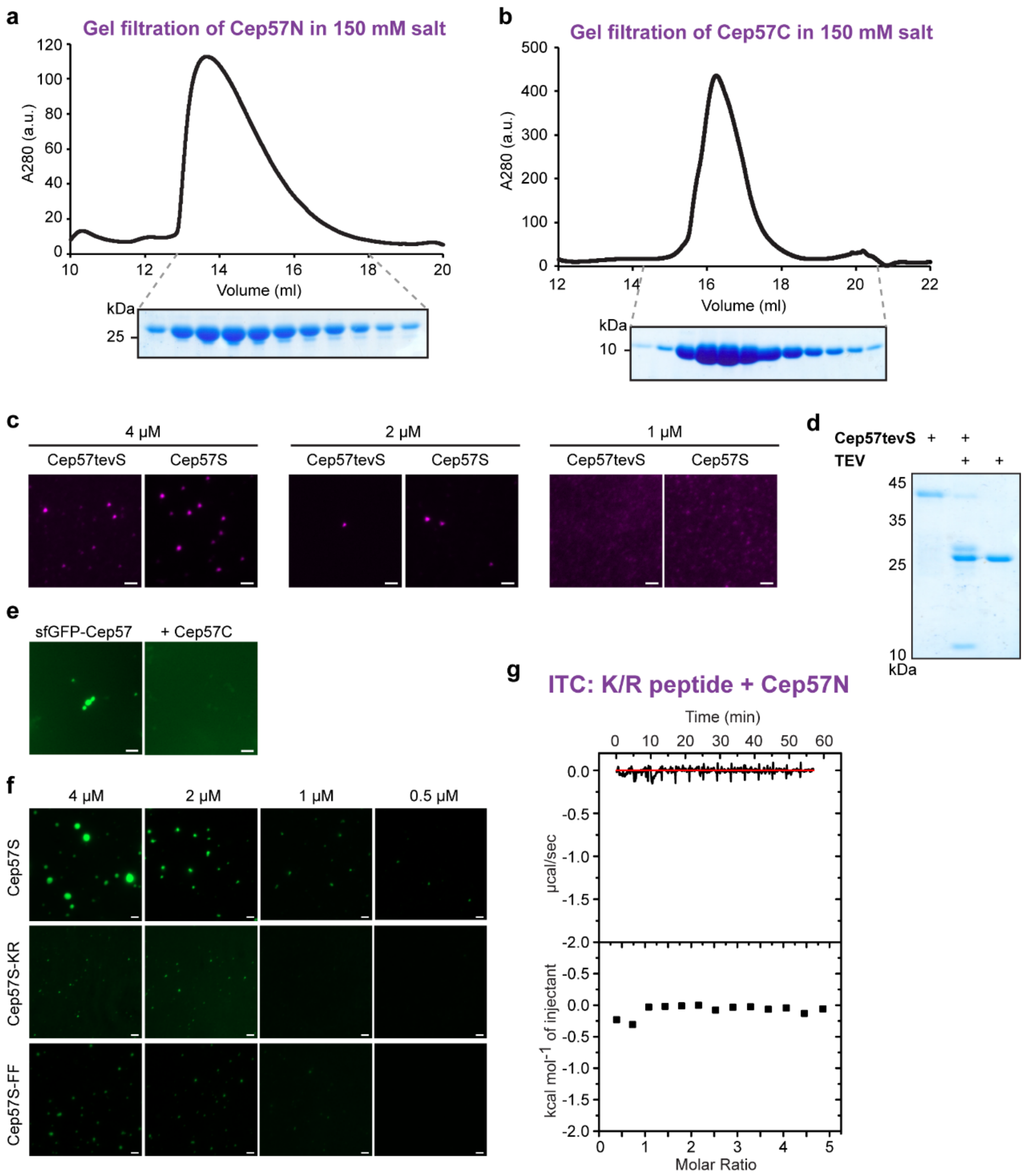

Figure S2. Cep57N or Cep57C alone cannot assemble higher-order complexes. a, b Gel filtration profiles of Cep57N and Cep57C in 150 mM salt. Gel filtration analysis of Cep57N (2.5 mg) and Cep57C (5 mg) by a Superdex 200 Increase 10/300 GL column. Peak fractions were examined by SDS-PAGE. **c** Cep57tevS and Cep57S exhibited similar potency toward LLPS**.** Cep57tevS and Cep57S condensates were formed in 200 mM NaCl at 22 ºC overnight before image acquisition. 5% of Cy5-Cep57S (magenta) was included. Scale bar, 2 μm. **d** SDS-PAGE analysis of Cep57tevS in the absence or presence of TEV protease. **e** TIRF images of 100 nM sfGFP-Cep57 in the presence of 10 μM Cep57C. Images were acquired after overnight incubation in the presence

of 5% PEG3350. Scale bar, 2 μm. **f** Critical concentration measurements for Cep57S, Cep57S-KR, and Cep57S-FF condensates formation. Condensates were formed in 200 mM NaCl at 22 °C overnight before image acquisition. Scale bar, 2 μm. **g** ITC measurement of 1 mM K/R-rich peptide (RRIKKKKSKPPEKKSSR) titrated to 44 μM of Cep57N.

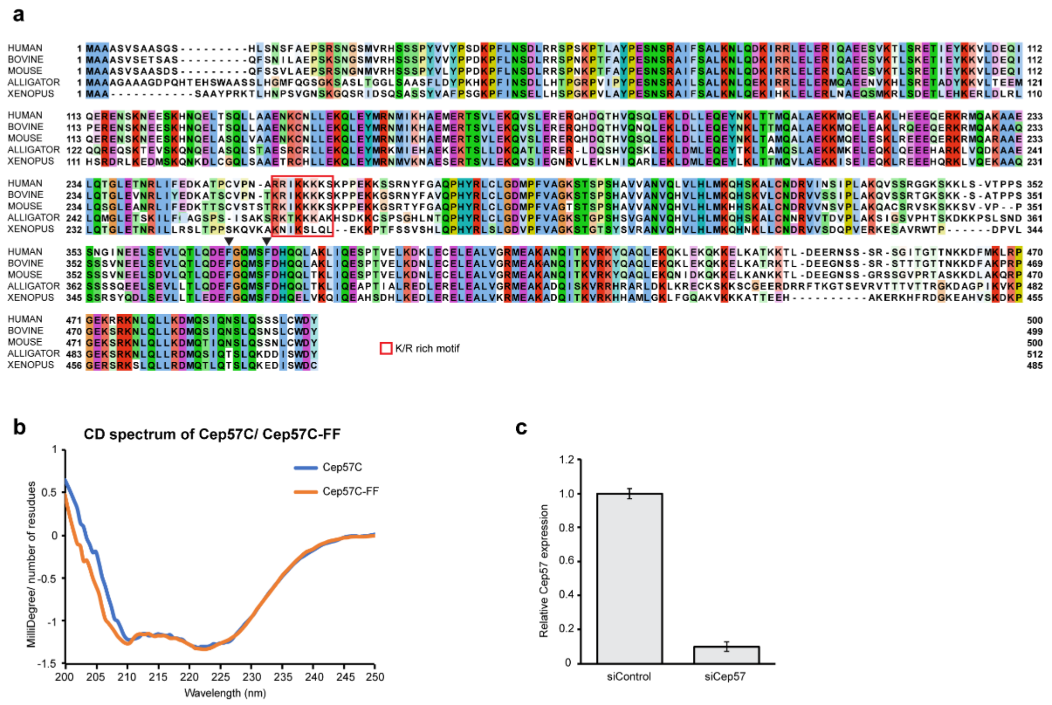

Figure S3. K/R rich motif and the C-terminal dimerization interface in Cep57 are conserved across species. a Sequence alignment of Cep57 among different species, aligned by ClustalX (1) and presented by Jalview (2). A red box marks the K/R rich motif. A solid triangle indicates the conserved F371 and F376. **b** Circular dichroism spectra of Cep57C and Cep57C-FF. Cep57C (4 μM) and Cep57C-FF (3 μM) were scanned in the far-UV range (200-250 nm) in 50 mM sodium phosphate pH 7.5. **c** Cep57 expression level 30 hours after siRNA transfection in HeLa cells examined by qPCR.

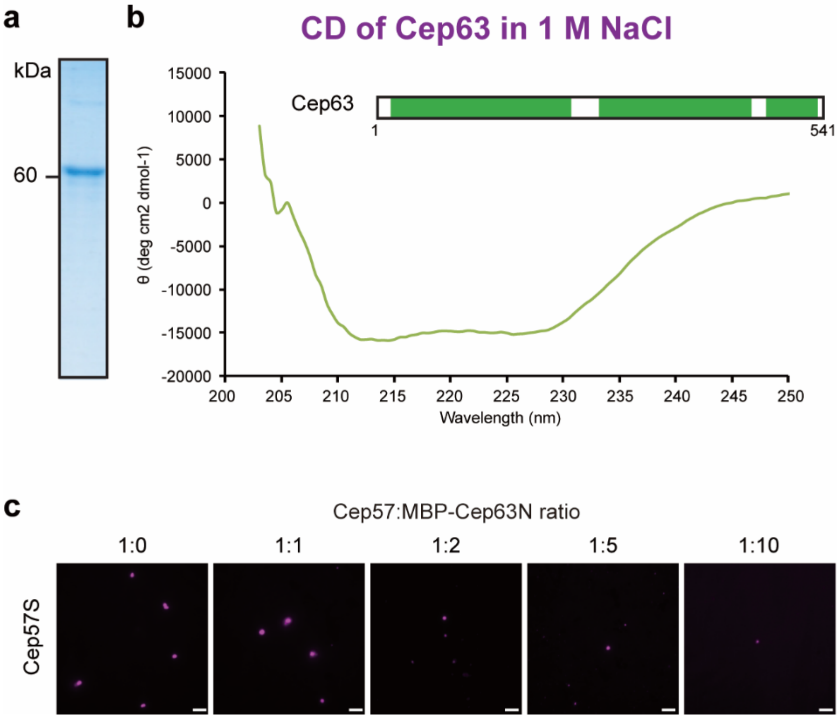

Figure S4. Cep63 negatively regulates the phase separation of Cep57S. a,b SDS-PAGE profile of purified Cep63 and the circular dichroism spectra. 11 μM of Cep63 was scanned in the far-UV range (200-250 nm) in buffer containing 20 mM Tris-HCl pH 8.0, 1M NaCl, 1 mM 2-mercaptoethanol, and 10% glycerol (w/v). **c** TIRF images of Cep57S condensates supplemented with MBP-Cep63N in different molar ratios. Cep57S concentration was fixed at 5 μM, including 5% of Cy5-Cep57S (magenta). Scale bar, 5 μm.

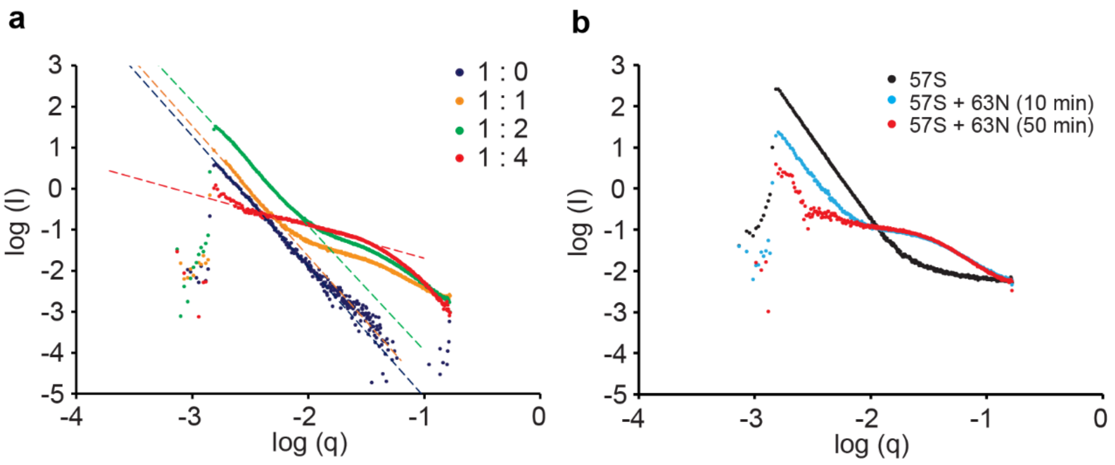

Figure S5. SAXS intensity of Cep57S in the presence of MBP-Cep63N. a SAXS profile of Cep57S fixed at 10 μM in the presence of MBP-Cep63N at indicated ratios (Cep57S:MBP-Cep63N). Proteins were incubated at 22 °C for 12 hours before the SAXS experiments. The dotted line represents the extrapolation of the linear regression of scattering data in the q range from 0.01 Å^{-1} to 0.002 Å^{-1} or 0.01 Å^{-1} to 0.006 Å^{-1} (for 1:1 only). Slopes for linear regression are -3.2 (1:0), -3.2 (1:1), -3.0 (1:2), and -0.8 (1:4). As MBP-Cep63N increased, the absolute value of the slope decreased, implying that the complexity of the structure decreased. **b** Time-dependent SAXS curve of Cep57S before (black), 10 minutes (blue), or 50 minutes (red) after supplement of MBP-Cep63N with a molar ratio 1:4 (Cep57S:MBP-Cep63N). All experiments were performed in the buffer containing 20 mM Tris-HCl pH 8.0, 200 mM NaCl, 10% glycerol, and 1 mM 2-mercaptoethanol.

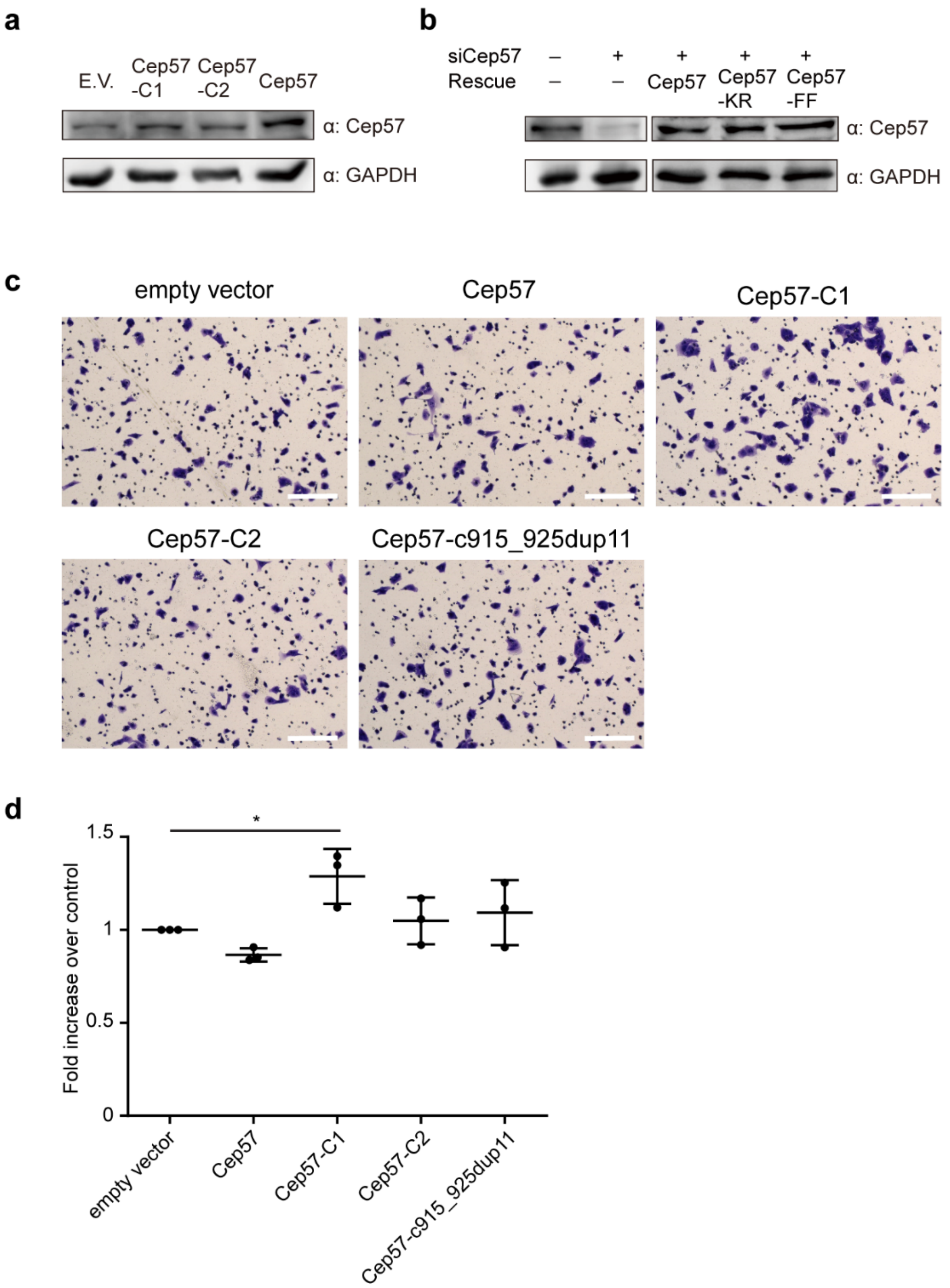

Figure S6. Overexpression of Cep57-C1 promoted cell motility. a Western blot of endogenous Cep57 of HeLa cells overexpressing the indicated constructs. **b** Western blot of Cep57 from HeLa cells treated with siRNA and transfected with the indicated constructs. Experiments in **a** and **b** are repeated twice. **c** Images of three regions were taken from duplicated inserts, and the numbers of cells were counted using ImageJ. Scale bars: 200 μm. **d** Number of migrated cells transfected with indicated constructs was plotted as fold increase over control (empty vector). Data represent means ± SD from 3 independent experiments. [*] $p<0.05$ by one-way ANOVA; Dunnett's multiple comparison test.

Supplementary Materials and Methods

Cloning and protein purification

All the constructs were cloned from the human cDNA library into a pCool2-His plasmid by standard PCR reactions, Gibson assembly, or T4 ligation for recombinant protein expression in the bacterial system. The QuickChange mutagenesis was used to create Cep57 mutations. For cell assays, Cep57 fragments were cloned into a pmCherry-N1 plasmid, a c-Myc vector derived from pmCherry-N1, or an HA vector by conventional PCR and Gibson assembly methods. All Cep57 and Cep63 constructs were purified by affinity chromatography, followed by gel filtration chromatography in 1M NaCl. SF9 cells overexpressing sfGFP-Cep57 were harvested after 50 hours of baculovirus infection, followed by tandem affinity purification for 6x His tag and twin-strep-tag® in buffer containing 20 mM Tris-HCl pH 8.0, 1 M NaCl, 1 mM 2-mercaptoethanol, 2 mM PMSF, and 10% glycerol (w/v). 300 mM of imidazole or 8 mM of dethiobiotin was supplemented to the described buffer for protein elution.

Isothermal titration calorimetry (ITC)

The polybasic LMN peptide was synthesized by Kelowna International Scientific Inc., and dissolved in ITC buffer (150 mM potassium phosphate pH 7.5, 1 mM 2-mercaptoethanol, and 10% glycerol (w/v)). All proteins and peptides were dialyzed against the ITC buffer at 4 °C overnight prior to ITC measurements. 200 μL of receptor was titrated by 40 μL of titrant for each measurement. The initial concentrations of titrants and receptors for different measurements are listed as the following (titrant + receptor): 400 μM Cep57C + 15 μM Cep57N; 170 μM Cep63N + 15 μM Cep57N; 1500 μM Cep57C + 50 μM LMN; 1 mM LMN + 44 μM Cep57N. The measurements were conducted by 14 injections with 500 rpm of stirring speed, and 240 seconds spacing time at 25°C or 15°C as listed in Table S1 (MicroCal iTC200, GE Healthcare). Curve fitting was accomplished in Origin® according to a single binding site model.

His-tag pull-down assay

Ni-NTA spin columns (Qiagen) were used to perform α/β-tubulin pull-down assay by Cep57S. Cep57S were incubated in the spin column for 30 min, followed by 3 cycles of re-binding at 4 °C. The column bound with Cep57S construct or MBP (0.5 mg input for each) was washed three times by binding buffer (1X PBS pH 8.0, 1 mM 2-mercaptoethanol, 20 mM imidazole, 0.1% Triton

X100, and 10% glycerol (w/v)). 0.1 mg of α/β-tubulin (Cytoskeleton, Inc) was then incubated in the column for 30 min, followed by 3 cycles of re-binding at 4 °C. After five washes, proteins were eluted in elution buffer (1X PBS pH 7.4, 850 mM NaCl, 1 mM 2-mercaptoethanol, 400 mM imidazole, and 10% glycerol (w/v)) and subjected to SDS-PAGE analysis.

Condensate and microtubule assembly assays

In vitro condensate assembly was conducted by diluting the concentrated protein samples with 5% of fluorophore-labeled samples into assay buffer (20 mM Tris-HCl pH 8.0, 1 mM 2-mercaptoethanol, 1 mM PMSF, and 10% glycerol (w/v)) to reach the target protein and salt concentrations for each experiment, and the condensates were settled on 3.5 cm glass culture dishes (Alpha Plus) for 4-6 hours (unless otherwise indicated) before imaging. For microtubule assembly assays, Cep57S condensates were prepared by incubation in BRB80 buffer (80 mM PIPES, 1 mM MgCl2, 1 mM EGTA) supplemented with 150 mM NaCl, 10% glycerol, and 5% PEG3350 at 22 °C. After 4 hours of incubation, Cep57S condensates were supplemented with 1 mM Mg/GTP, 0.5 μM Oregon Green 488 taxol (Thermo Fisher Scientific), and α/β-tubulin (Cytoskeleton, Inc). Before imaging, the mixture was transferred to a 3.5 cm glass culture dish (Alpha Plus). The control group, spontaneous microtubule nucleation, followed the same procedures without Cep57S. For the tubulin concentration assay, Cep57S condensates were prepared in the same condition as the microtubule nucleation assay. After 4 hours of incubation, 66 μM nocodazole (Selleckchem) was added to inhibit microtubule nucleation before supplementing 5 μM Cy5-tubulins (5% labeled). Each assay was repeated three times.

Image acquisition and analysis

Images of Cep57S condensates in Fig. 1e, FRAP analysis (Fig. 1i, j), and the time-series record of Cep63 recruitment to Cep57S condensates (Fig. 5b) were acquired by a ZEISS LSM 800 with a 63x Plan-Apochromat DIC oil objective. Cell images for Fig. 6e were acquired by a Leica DMI6000 microscope equipped with an HCX PL Apo 63x/1.4 Oil objective and an Andor Luca R EMCCD camera; others were acquired by ZEISS Axio Observer Z1 Apotome with a 63x Plan-Apochromat DIC oil objective. *In vitro* reconstitution was imaged by ZEISS Axio Observer Z1 TIRF with a 100x Plan-Apochromat DIC oil objective. TIRF, Apotome, and confocal images were processed by ZEN blue 3.1. The size and intensity of the condensates were quantified by applying “image threshold” and “particle analysis” functions on FIJI (3). The partition

coefficient was defined as the condensate intensity divided by the averaged solution phase intensity from three ROIs. All assembly assays were repeated three times.

Fluorescence recovery after photobleaching (FRAP) analysis

For FRAP analysis, the Cep57S condensates were incubated on the α-plus 3.5 cm glass culture dish for either 0.5-1 hour or 4 hours in the presence of 5% Dylight488-labeled proteins. The FRAP measurements were conducted by "photobleaching" and "time series" functions of ZEN blue 3.1 on ZEISS LSM 800, using a 63x oil objective under 3.5x crop and maximum pinhole. The degree of photobleaching was set to 50%. After photobleaching, the images were taken every 15 seconds for young droplets and 30 seconds for old droplets, and the intensity of the bleached region was recorded and normalized by the changes of the nearby condensates with similar sizes accounting for bleaching during image acquisition. Data fitting was performed by Prism8 using a one-phase association model (% recovery = Plateau * (1 – $e^{-Time/Tau}$)).

Size Exclusion Chromatography Multi-Angle Light Scattering (SEC-MALS)

SEC-MALS experiments were performed by using size-exclusion column Superdex 200 Increase 5/150 GL (Cytiva) equipped with 3 detectors: an Agilent 1260 DAD (UV), a Wyatt DAWN-HELEOS-II detector (MALS), and a Wyatt Optilab T-Rex differential refractive index detector (RI). Superdex 200 column was equilibrated with a buffer containing 20 mM HEPES pH 7.5, 150 mM NaCl, and 1 mM DTT. 100 μl of Cep57C (1 mg/ml) or 10 μl of Cep57C-FF (4 mg/ml) were loaded onto the column using an auto-sample injection method with the flow rate 0.2 ml/min at 10 °C. Data were analyzed by Wyatt Astra software (version 7.3.1).

Size Exclusion Chromatography coupled to Small- and Wide-Angle X-ray Scattering (SEC-SWAXS)

SEC-SWAXS experiments were performed at the TPS 13A BioSWAXS beamline of the National Synchrotron Radiation Research Center, Taiwan (4, 5). The beamline is equipped with two in-vacuum Eiger X 9M and X 1M detectors for SWAXS and an in-line HPLC unit (Agilent 1260 series). A Bio SEC-3 silica-based column (pore size 300 Å, Agilent) was equilibrated with a buffer containing 20 mM Tris-HCl pH 8.0, 150 mM/1 M NaCl, and 1 mM 2-mercaptoethanol before SEC-SWAXS measurements. The sample solution of 100 μl of Cep57N at 5 mg/ml was loaded onto the column with a flow rate of

0.35 ml/min at 10 °C. The eluate from the SEC was directed to the quartz capillary (2 mm dia. and a wall thickness of 20 μm) of the SEC-SWAXS system for X-ray exposure with a 15-keV beam (dimensions 260 μm by 350 μm). The scattering data were collected continuously with 2 s per frame (with 0.2 s between frames) over the elution peak. The frame data of well-overlapped SAXS profiles were averaged and subtracted with buffer scattering using the TPS 13A SWAXS Data Reduction Kit (Ver. 3.6), and analyzed using ATSAS 3.1.3 (6).

Protein crystallography

His-Cep57C was concentrated at 10 mg/ml for crystallization. The crystal was grown in a screen containing 0.1 M Sodium chloride, 0.1 M HEPES pH 7.5, 1.6 M Ammonium sulfate from HRII-112 (Hampton Research) at 22 °C by hanging drop vapor diffusion method. The X-ray diffraction data were collected at 100 K at beamline TLS 13B, NSRRC, Taiwan, equipped with an ADSC Quantum-315R CCD Area Detector. The X-ray wavelength was 1.0000 Å. Data were processed by HKL2000 (7). The crystal structure of Cep57C was solved by the molecular replacement program Phaser-MR in Phenix (8) using the previously determined structure (PDB ID: 4L0R) as the search model. Structural refinement and model building were carried out using Phenix (8) and Coot (9) iteratively. The structure was refined to 2.10 Å resolution with R_{work}/R_{free} of 0.238/0.257 and all residues (100%) in the Ramachandran favored regions. Compared to the existing model in the PDB, our structure has a higher resolution and better quality. The structure display was created by PyMOL.

Microtubule regrowth assay

Transient transfection of siRNA (MDBio, Inc) and plasmid DNA were sequentially carried out by TransIT-X2 (Mirus Bio) to HeLa cells after 24 hours of seeding. After 40 hours of transfection, microtubule depolymerization was initiated by a 1-hour cold treatment in the presence of 16.6 μM nocodazole. After three washes (2, 2, 5 min), microtubule re-assembly was initiated by incubating cells in the warm (37°C) medium for 2 minutes, followed by methanol fixation. Standard immunostaining procedures were carried out using the following primary antibodies: anti-pericentrin (Abcam), anti-α-tubulin (Santa Cruz); and the following secondary antibodies: Alexa Fluro 488-conjugated goat anti-mouse IgG (Invitrogen), Alexa Fluro 594-conjugated donkey anti-rabbit IgG (Invitrogen). DNA staining was carried out by 300 nM DAPI (Invitrogen). Cell assays were performed three times.

Centrosomal Cep57 perturbation assay

Transient transfection to HeLa cells was carried out by TransIT-X2 (Mirus Bio) after 12-24 hours of seeding. Cells were fixed after 18-24 hours of transfection with ice-cold methanol for 10 min. Standard immunostaining procedures were carried out using the following primary antibodies: anti-Cep57 (Invitrogen, immunogen: residues 118-226), anti-centrin2 (Merck); and the following secondary antibodies: Alexa Fluro 488-conjugated goat anti-mouse IgG (Invitrogen), Alexa Fluro 594-conjugated donkey anti-rabbit IgG (Invitrogen). DNA staining was carried out with 300 nM DAPI (Invitrogen). Cell assays were performed three times.

Centrosome amplification assay

Transient transfection to Hela cells was carried out upon seeding by FuGENE®HD (Promega) for 48 hours, followed by a 2 mM thymidine treatment (Sigma-Aldrich) for 16 hours, and a 12-hour treatment of 7 mM RO3306 (Sigma-Aldrich). Cells were released from the G2/M block by washing out RO3306. After 20 minutes of incubation at 37 °C, cells were fixed with ice-cold methanol for 10 min. Standard immunostaining procedures were carried out using the following primary antibodies: anti-pericentrin (Abcam), anti-centrin2 (Merck); and the following secondary antibodies: Alexa Fluro 488-conjugated goat anti-mouse IgG (Invitrogen), Alexa Fluro 594-conjugated donkey anti-rabbit IgG (Invitrogen). DNA staining was carried out by 300 nM DAPI (Invitrogen). Cell assays were performed four times.

Cep57 knockdown and rescue assay

Transient transection of siRNA (MDBio, Inc) and plasmid DNA were sequentially carried out by TransIT-X2 (Mirus Bio) to HeLa cells after 24 hours of seeding. Cell synchronization was carried out after 14 hours of plasmid transfection following the same procedures as mentioned in the centrosome amplification assay. Standard immunostaining procedures were carried out using the following primary antibodies: anti-pericentrin (Abcam), anti-centrin2 (Merck); and the following secondary antibodies: Alexa Fluro 488-conjugated goat anti-mouse IgG (Invitrogen), Alexa Fluro 594-conjugated donkey anti-rabbit IgG (Invitrogen). DNA staining was carried out with 300 nM DAPI (Invitrogen). Cell assays were performed five times.

SI Appendix References

1. M. A.Larkin, *et al.*, Clustal W and Clustal X version 2.0. *Bioinformatics* **23**, 2947–2948, (2007).
2. A. M.Waterhouse, J. B.Procter, D. M. A.Martin, M.Clamp, G. J.Barton, Jalview Version 2-A multiple sequence alignment editor and analysis workbench. *Bioinformatics* **25**, 1189–1191, (2009).
3. J.Schindelin, *et al.*, Fiji: An open-source platform for biological-image analysis. *Nat. Methods* **9**, 676–682, (2012).
4. D. G.Liu, *et al.*, Optical design and performance of the biological small-Angle X-ray scattering beamline at the Taiwan Photon Source. *J. Synchrotron Radiat.* **28**, 1954–1965, (2021).
5. O.Shih, *et al.*, Performance of the new biological small- and wide-angle X-ray scattering beamline 13A at the Taiwan Photon Source. *J. Appl. Crystallogr.* **55**, 340–352, (2022).
6. K.Manalastas-Cantos, *et al.*, ATSAS 3.0: expanded functionality and new tools for small-angle scattering data analysis. *J. Appl. Crystallogr.* **54**, 343–355, (2021).
7. Z.Otwinowski, W.Minor, Processing of X-ray diffraction data collected in oscillation mode. *Methods Enzymol.* **276**, 307–326, (1997).
8. P. D.Adams, *et al.*, PHENIX: A comprehensive Python-based system for macromolecular structure solution. *Acta Crystallogr. Sect. D Biol. Crystallogr.* **66**, 213–221, (2010).
9. P.Emsley, K.Cowtan, Coot: Model-building tools for molecular graphics. *Acta Crystallogr. Sect. D Biol. Crystallogr.* **60**, 2126–2132, (2004).
